# HDAC4 preserves skeletal muscle structure following long-term denervation by mediating distinct cellular responses

**DOI:** 10.1186/s13395-018-0153-2

**Published:** 2018-02-24

**Authors:** Eva Pigna, Alessandra Renzini, Emanuela Greco, Elena Simonazzi, Stefania Fulle, Rosa Mancinelli, Viviana Moresi, Sergio Adamo

**Affiliations:** 1 DAHFMO Unit of Histology and Medical Embryology, Interuniversity Institute of Myology, Sapienza University of Rome, Rome, Italy; 2 Department of Neuroscience Imaging and Clinical Sciences-Section of Physiology and Physiopathology, University “G. d’Annunzio” Chieti-Pescara, Chieti, Italy

**Keywords:** HDAC4, Oxidative stress, Denervation, UPS, Autophagy, HDAC inhibitors

## Abstract

**Background:**

Denervation triggers numerous molecular responses in skeletal muscle, including the activation of catabolic pathways and oxidative stress, leading to progressive muscle atrophy. Histone deacetylase 4 (HDAC4) mediates skeletal muscle response to denervation, suggesting the use of HDAC inhibitors as a therapeutic approach to neurogenic muscle atrophy. However, the effects of HDAC4 inhibition in skeletal muscle in response to long-term denervation have not been described yet.

**Methods:**

To further study HDAC4 functions in response to denervation, we analyzed mutant mice in which HDAC4 is specifically deleted in skeletal muscle.

**Results:**

After an initial phase of resistance to neurogenic muscle atrophy, skeletal muscle with a deletion of HDAC4 lost structural integrity after 4 weeks of denervation. Deletion of HDAC4 impaired the activation of the ubiquitin-proteasome system, delayed the autophagic response, and dampened the OS response in skeletal muscle. Inhibition of the ubiquitin-proteasome system or the autophagic response, if on the one hand, conferred resistance to neurogenic muscle atrophy; on the other hand, induced loss of muscle integrity and inflammation in mice lacking HDAC4 in skeletal muscle. Moreover, treatment with the antioxidant drug Trolox prevented loss of muscle integrity and inflammation in in mice lacking HDAC4 in skeletal muscle, despite the resistance to neurogenic muscle atrophy.

**Conclusions:**

These results reveal new functions of HDAC4 in mediating skeletal muscle response to denervation and lead us to propose the combined use of HDAC inhibitors and antioxidant drugs to treat neurogenic muscle atrophy.

**Electronic supplementary material:**

The online version of this article (10.1186/s13395-018-0153-2) contains supplementary material, which is available to authorized users.

## Background

Loss of muscle innervation occurs in numerous disorders, such as in amyotrophic lateral sclerosis (ALS) or in spinal muscular atrophy (SMA), but also following viral infections or during aging, accounting for a drastic loss of muscle mass. Different molecular mechanisms, including the catabolic pathways—e.g., the ubiquitin-proteasome system and autophagy—and oxidative stress (OS), which are normally involved in the maintenance of muscle homeostasis, are activated following muscle denervation, thus contributing to muscle atrophy [1–3]. Degradation through the ubiquitin-proteasome system (UPS) is initiated by the sequential addition of ubiquitin molecules to target proteins, catalyzed by E1-, E2-, and E3-ubiquitinating enzymes. The polyubiquitinated proteins are then recognized and processed by the proteasome, a multi-catalytic proteolytic complex. Two key factors for inducing skeletal muscle atrophy are the E3-ubiquitin ligases atrogin1 and MuRF1. Consistently, mice lacking either factor are resistant to neurogenic muscle atrophy [4]. Another catabolic pathway activated in skeletal muscle following denervation is autophagy [5, 6]. Damaged or dysfunctional intracellular components are polyubiquitinated and included into double-membrane structures, the autophagosomes, to be degraded by autophagy. Several autophagy-related (Atg) genes are induced and participate in the formation of the autophagosomes, while the cytosolic LC3bI is converted to LC3bII, allowing its association with the developing autophagosome membrane. The chaperone protein p62 binds to polyubiquitinated proteins and to LC3b or Gabarapl1, thus guiding the cargo into autophagosomes to be digested [7–9]. Since p62 itself is degraded after the fusion of autophagosomes with lysosomes, it is commonly used as a marker for the evaluation of the autophagic flux [10]. Although initially considered as a non-selective degradation pathway, autophagy plays a role in the selective removal of specific organelles, such as mitochondria (via mitophagy) or protein aggregates. After depolarization of the mitochondrial membrane or accumulation of mitochondrial misfolded proteins, PINK1 is stabilized at the outer mitochondrial membrane, where it phosphorylates and activates Park2 [11, 12], targeting mitochondria for removal by autophagy [13, 14]. For a long time, UPS and autophagy were considered independent pathways. However, several proteins, e.g., α-synuclein or aggregate-prone proteins, are known to share these two degradative pathways [15–18], indicating that UPS and autophagy may be interchangeable, depending on cellular necessity. Indeed, the inhibition of either process has been shown to drive the activation of the other in physiological contexts [19–23].

Besides activating the catabolic pathways, denervation induces increasing levels of OS in skeletal muscle [24]. Oxidants damage cellular components and cause cellular dysfunctions. To cope with OS, organisms have developed multiple defense mechanisms to alleviate oxidative damage, including antioxidants enzymes, molecular chaperones, and proteolytic systems. Indeed, both autophagy and UPS are activated in response to OS to remove damaged proteins [25, 26].

Epigenetic processes fine-tune cellular responses to different stresses, including denervation [26–29]. Class II histone deacetylases (HDACs) are involved in the transcriptional activation of the E3-ubiquitin ligases atrogin1 and MuRF1 and the activation of the MAPK-AP1 axis following denervation [30, 31]; while Sirt1, HDAC6, HDAC1, and HDAC2 have been shown to regulate autophagy and therefore the maintenance of muscle homeostasis [32–34].

HDAC4 is a member of class IIa HDACs, rapidly induced in skeletal muscle upon denervation and able to mediate skeletal muscle response. Following denervation, HDAC4 indirectly regulates myogenin expression, thereby connecting neuronal activity to skeletal muscle transcriptional reprogramming of the neuromuscular junctions and compensatory reinnervation [35]. Considering its crucial role, HDAC4 has been proposed as a potential therapeutic target for diseases characterized by neurogenic muscle atrophy, such as ALS or SMA, or for sarcopenia [36–39]. Any pharmacological treatment with HDAC4 inhibitors for these conditions should be continued for months or years. However, the effects of HDAC4 inhibition in skeletal muscle following long-term denervation have not been investigated yet.

To delineate the role of HDAC4 in skeletal muscle in a condition of long-term denervation, we cut the sciatic nerve and analyzed skeletal muscle-specific HDAC4 mutant mice (hereafter referred to as HDAC4mKO mice) over time. We demonstrate that inhibition of HDAC4 leads to loss of muscle integrity and inflammation upon long-term denervation, because of the impairment in the activation of the UPS and a delay in triggering the autophagic pathway. Moreover, HDAC4mKO mice showed altered OS response, and an antioxidant treatment reduced loss of muscle integrity and inflammation in HDAC4mKO, preserving resistance to neurogenic muscle atrophy. These findings reveal that inhibition of HDAC4 could be detrimental for skeletal muscle in a condition of long-term denervation. However, the combined use of antioxidant drugs and HDAC inhibitors may prove useful in the treatment of ALS, SMA, and sarcopenia.

## Methods

### Mice

HDAC4 conditional mutant mice were previously generated and described [40]. Adult (10 weeks old) HDAC4^fl/fl^myogenin;Cre (HDAC4mKO) and HDAC4^fl/fl^ (control mice) female mice were used throughout the experiments. Mice were treated in strict accordance with the guidelines of the Institutional Animal Care and Use Committee and to national and European legislation, throughout the experiments. Animal protocols were approved by the Italian Ministry of Health (authorizations # 244/2013; # 80/2014-B; # 138/2016-PR).

#### Denervation

In anesthetized adult mice, the sciatic nerve of the left limb was cut, and a 3 mm piece was excised. The right leg remained innervated and was used as control. Mice were sacrificed at indicated time points for histological and molecular analyses.

#### Colchicine administration

To assess autophagic flux, mice were treated with either 0.4 mg/kg/day colchicine (Sigma-Aldrich) or an equal volume of vehicle (water), by daily intraperitoneal (i.p.) injections, for 4 days prior to sacrifice, as previously described [41].

#### Methylene blue administration

To trigger proteasome activity, mice were treated with 25 mg methylene blue (Panreac AppliChem) per 100 g of chow, starting from the day of denervation [42].

#### Intermittent fasting

Chow was removed from the cages for 24 h, twice a week, for 4 weeks, starting from the day of denervation.

#### Trolox

Mice were treated 5 days a week, starting from the day of denervation, by i.p. injections of 15 mg/kg Trolox (Sigma) in 40% NaOH in physiologic solution (vehicle) or an equal volume of vehicle as controls, for 4 weeks.

#### DNA delivery by electroporation

In anesthetized mice, *Tibialis anterior* (TA) muscles were exposed, injected with 25 μl of DNA, containing 8.4 μg of dsRED and 20 micrograms of Ub-G76V-YFP plasmids, in a 5% mannitol solution, and immediately subjected to electroporation. 3 days later, mice were subjected to denervation and then analyzed 2 weeks following the electroporation.

### Histological analyses

TA muscles were dissected, embedded in tissue freezing medium (Leica, Wetzlar, Germany), and frozen in isopentane pre-cooled with liquid nitrogen. Cryosections (8 μm) were obtained by using a Leica cryostat. Hematoxylin and eosin (H&E) and Masson’s trichrome staining (Sigma-Aldrich) were performed according to the manufacturer’s instructions.

Dihydroethidium (DHE) staining was performed on transverse cryosections of TA muscles. Unfixed cryosections were incubated with 0.002 mM DHE (Molecular Probes D1168) in DMSO for 30 min at 37 °C. Coverslips were mounted with 60% glycerol in Tris HCl 0.2 M pH 9.3.

For immune-histochemical analyses, cryosections were fixed in 4% paraformaldehyde for 10 min at room temperature, then washed and blocked with 1% BSA (Sigma, St. Louis, MO) for 30 min or with 5% Goat serum for 1 h. Samples were then incubated with one of the following antibodies, diluted in 1% BSA: 1:100 rabbit polyclonal anti-laminin antibody (Sigma), 1:500 mouse monoclonal anti-CD68 (AbD Serotec), 1:100 rabbit polyclonal anti-dystrophin (Abcam), 1:50 rabbit polyclonal anti-alpha-dystroglycan (Santa Cruz), overnight at 4 °C. To detect the primary antibody, incubation with a 1:500 dilution of anti-rabbit-Alexa 488 or anti-Rat-Alexa 488 (Life Technologies) secondary antibodies in 1% BSA for 1 h at room temperature was performed. For IgG staining, after blocking, muscles were incubated with a 1:500 dilution in 1% BSA of anti-mouse-Alexa 555 (Life Technologies) secondary antibody, for 1 h at room temperature. 0.5 μg/ml Hoechst 33,342 (Sigma) was used to stain nuclei.

#### Morphometric analyses

Myofiber cross-sectional area, CD68^+^ area, and connective tissue were quantified by using ImageJ software. For myofibers distribution analyses, about 600 fibers were counted for each sample.

### RNA extraction and real-time PCR

Total RNA was isolated and purified from 30 to 50 mg of TA muscles by using Trizol (Invitrogen), following the manufacturer’s protocol. One microgram of total RNA was converted to cDNA by using the PrimeScript™ RT reagent Kit (Takara). Real-time PCR was performed with the SDS-ABI Prism 7500 (Applied Biosystem), by using the Sybr Green reaction mix (Applied Biosystem). Primers are listed in Table 1.

**Table 1:** Primer used for real-time PCR

Gene	Forward	Reverse
Atg7	GCCTAACACAGATGCTGCAA	TGCTCTTAAACCGAGGCTGT
Gabarapl1	GTGCCGGTCATCGTGGA	TCCTCGTGGTTGTCCTCA
p62	CCCAGTGTCTTGGCATTCTT	AGGGAAAGCAGAGGAAGCTC
Gsr1	AAT TGG CGT GTT ATT AAG GAA AAG C	TCT ATA TGG GAC TTG GTG AGA TTG T
Gstp1	ATG ACT ATG TGA AGG CAC TG	AGG TTC ACG TAC TCA GGG GA
NQO1	CAT TCT GAA AGG CTG GTT TGA	CTA GCT TTG ATC TGG TTG TCA G
Catalase	CCT CCT CGT TCA AGA TGT GGT TTT C	CCT CCT CGT TCA AGA TGT GGT TTT C
Gapdh	ACCCAGAAGACTGTGGATGG	CACATTGGGGGTAGGAACAC

### Protein extraction and western blot analyses

TA muscles were dissected, minced, and homogenized in lysis buffer (50 Mm Tris HCl pH 7.4, 1 mM EDTA, 150 Mm NaCl, 1% Triton) supplemented with protease and phosphatase inhibitors. Proteins (30–50 μg) were separated by SDS-PAGE and transferred to PVDF membrane (Invitrogen). Unspecific binding was blocked with 5% not fat dry milk in TBST buffer (20 mM Tris-HCl pH 7.6, 137 mM NaCl, 0.5% Tween 20) then membranes were incubated overnight at 4 °C, with primary antibody diluted in 5% BSA (Sigma) in TBST. After washing in TBST, membranes were incubated with secondary antibodies HRP-conjugate (Bio-Rad 170-6515 or 170-6516) and signals were detected by luminol-enhancer solution (Cyanagen). Images were acquired using films or ChemiDoc MP imaging system (Bio-Rad). Densitometric analyses were performed by measuring band intensity for each sample using ImageJ software. The following primary antibodies were used: Gapdh (Santa Cruz sc-32,233), LC3b (Cell Signaling 2775), p62 (Sigma P0067), Gp91phox (BD Transduction Laboratories), MF20 (Developmental Studies Hybridoma Bank).

### Proteasome assay

TA muscles were dissected, minced, and homogenized in western blot lysis buffer. 10 μg of freshly isolated protein extracts were used for measuring proteasome activity, by using the CHEMICON Proteasome Activity Assay Kit (APT280, Millipore), following manufacturer’s instructions. Briefly, the extracts were incubated (2 h at 37 °C) with a labeled substrate, LLVY-7-amino-4-methylcoumarin, and the cleavage activity was monitored by detection of the free fluorophore 7-amino-4-methylcoumarin using a fluorescence plate reader (Infinite F200 PRO, TECAN) at 360/460 nm.

### Antioxidant enzymes activities

Muscles were homogenized in 100 mM Na-phosphate buffer pH 7.0 or pH 6.5 depending on the assay, with protease inhibitors cocktail (Sigma), and centrifuged at 13000 rpm for 15 min at 4 °C. Cytosol proteins were measured on the resulting supernatant according to the Bradford method. Glutathione S-transferase activity was determined by using 1-chloro-2-4-dinitrobenzene (CDNB) as substrate. The assay was performed at 340–410 nm (ε = 9.6 mM^− 1^ cm^− 1^) in a final volume of 1 ml containing 100 mM Na-phosphate buffer pH 6.5, 1 mM CDNB, 1 mM reduced glutathione and 50 μg of protein lysate. Glutathione reductase activity was measured by the rate of decrease in absorbance induced by NADPH oxidation, at 340 nm (ε = − 6.22 mM^− 1^ cm^− 1^). The assay mixture contained, in a final volume of 1 ml, 100 mM Na-phosphate buffer pH 7.0, 1 mM glutathione disulfide, 60 μM NADPH, and 100 mg of protein lysate.

### Dichlorodihydrofluorescein assay

Total free radical presence (reactive oxygen species and reactive nitrogen species) were quantified on muscle extracts by using OxiSelect Kit from Cell Biolabs, following the manufacturer’s instructions.

### Statistics

Statistical significance was determined by using two-way analysis of variance (ANOVA) followed by Tukey’s HSD as a post hoc test. All values are expressed as mean ± standard error of the mean (SEM). VassarStats, a statistical computation website available at http://vassarstats.net/, was used for the statistical analyses.

## Results

### Skeletal muscles of HDAC4mKO mice lost structural integrity following long-term denervation

To delineate the role of HDAC4 in skeletal muscle with a genetic approach following long-term denervation, the sciatic nerve of HDAC4mKO and control mice was cut, and TA muscles were analyzed over time. To reduce the number of experimental animals, contralateral, non-denervated muscles were used as controls [24]. Muscle mass evaluation showed that HDAC4mKO mice lost significantly less TA weight if compared with control mice, for the first 2 weeks after denervation, as previously published [31]. Interestingly, at later times, the resistance to neurogenic muscle atrophy displayed by the HDAC4mKO mice was lost: indeed, by the fourth week after denervation, the loss of muscle mass with respect to contralateral muscles was similar (approximately 40%) for HDAC4mKO and control mice (Additional file 1: Figure S1a). Immunostaining for laminin of TA cross-sections clearly delineated a decrease of muscle fiber size in control mice upon denervation, indicative of muscle atrophy (Additional file 1: Figure S1b). In contrast, the decrease in fiber size was less evident in the *HDAC4mKO* denervated muscles, even 4 weeks following denervation (Additional file 1: Figure S1b).

**Fig. 1:**
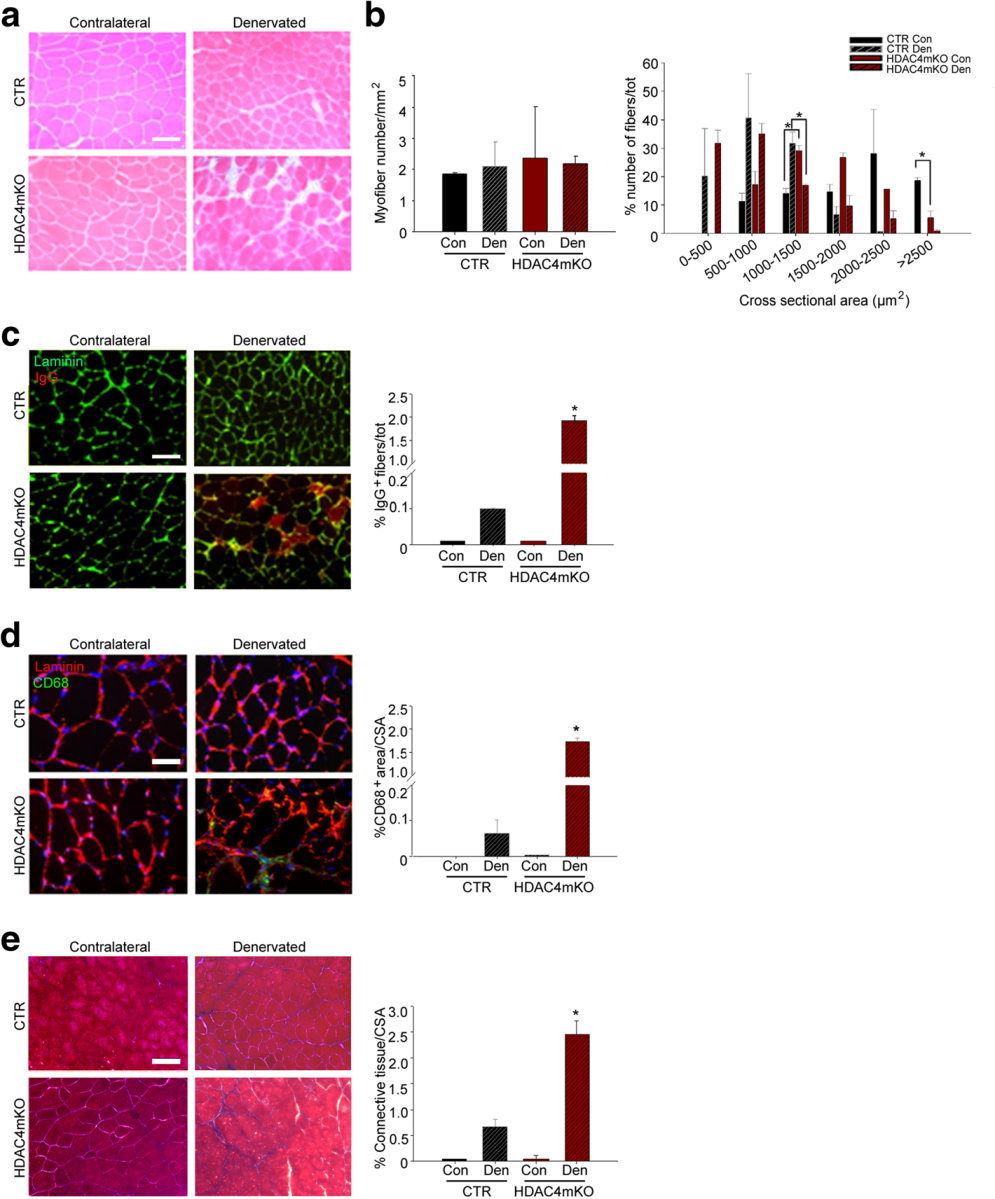
Skeletal muscles lacking HDAC4 shows compromised integrity following long-term denervation. **a** Hematoxylin and eosin staining of control (CTR) and HDAC4mKO TA muscles following 4 weeks of denervation. Scale bar = 50 μm. **b** Number of myofibers in CTR and HDAC4mKO contralateral and denervated muscles, 4 weeks after denervation. *n* = 3. Distribution analysis of fiber cross-sectional area of control (CTR) and HDAC4mKO mice, following 4 weeks of denervation. Data are shown as mean ± SEM; *n* = 3. Two-way ANOVA revealed an effect of genotype (*F* = 110.27; df 1; *p* = 0.001 for > 2500 μm^2^ class), an effect of denervation (*F* = 16.93; df 1; *p* = 0.009 for 500–1000 μm^2^ class; *F* = 390.97; df 1; *p* = 0.001 for 1500–2000 μm^2^ class and *F* = 18.5; df 1; *p* = 0.007 for > 2500 μm^2^ class) and an interaction (*F* = 104.35; df 1; *p* = 0.001 for 1000–1500 μm^2^ class and *F* = 139.33; df 1; *p* = 0.001 for > 2500 μm^2^ class); *p < 0.05 by Tukey’s HSD test. **c** Representative images for laminin (green) and IgG staining (red) of control (CTR) and HDAC4mKO TA muscles and relative quantification, following 1 week of denervation. Scale bar = 50 μm. *n* = 3; two-way ANOVA **s**howed an interaction (*F* = 8.41; df 1; *p* = 0.017); *p < 0.05 by Tukey’s HSD test. **d** Immunostaining for CD68 (green) and laminin (red) of control and HDAC4mKO TA muscles and relative quantification, following 1 week of denervation. Scale bar = 25 μm. *n* = 3; two-way ANOVA showed an interaction (*F* = 9.77; df 1; *p* = 0.012); *p < 0.05 by Tukey’s HSD test. **e** Masson’s trichrome staining of control (CTR) and HDAC4mKO TA muscles and quantification of connective tissue, following 4 weeks of denervation. Scale bar = 25 μm. *n* = 3; two-way ANOVA **s**howed an interaction (*F* = 39.42; df 1; *p* = 0.0004); *p < 0.05 by Tukey’s HSD test.

We further analyzed muscle histology and observed that, while denervation induced a reduction of fiber size in control mice, denervated HDAC4mKO muscles lost their structural integrity, showing infiltrated mononucleated cells and fibers with heterogeneous sizes (Fig. 1a). Loss of muscle mass could depend on a decrease in myofiber number and/or myofiber size. Therefore, we quantified the myofiber number in each condition and no significant differences were detected among samples (Fig. 1b). Morphometric and statistical analyses of the myofiber cross-sectional area (CSA) distribution confirmed a significant effect of denervation in 500–1000, 1500–2000, and > 2500 square micron classes (Fig. 1b). Moreover, the analyses revealed a significant higher number of smaller myofibers (1000–1500 μm²) in contralateral HDAC4mKO muscles respect to contralateral controls, at the expenses of the large ones (> 2500 μm²). Conversely, denervated HDAC4mKO muscles exhibited a significantly lower number of small myofibers (1000–1500 μm²) with respect to denervated control muscles (Fig. 1b).

To further characterize the HDAC4mKO phenotype after 4 weeks of denervation, IgG staining was performed and IgG^+^ fibers were quantified, detecting a significant increase in the presence of necrotic fibers in HDAC4mKO muscles following denervation, a finding virtually absent in contralateral muscles and poorly present in denervated control muscles (Fig. 1c). Since compromised fiber permeability is often associated with disruption of the dystrophin-glycoprotein complex (DGC), the expression and localization of two components of the DGC were evaluated in HDAC4mKO and control mice, following 4 weeks of denervation. No obvious differences in dystrophin or alpha-dystroglycan expression pattern were observed by immunofluorescence analyses (Additional file 1: Figure S2). The infiltrating mononuclear cells observed in denervated HDAC4mKO muscles were prevalently macrophages, as demonstrated by immunofluorescence and quantitative analyses for CD68 (Fig. 1d). Moreover, connective tissue was labeled by trichrome staining and quantified among samples. A significant accumulation of fibrotic tissue was registered only in denervated HDAC4mKO following 4 weeks of denervation (Fig. 1e).

### HDAC4 mediates the activation of UPS and autophagy in skeletal muscle upon denervation

**Fig. 2:**
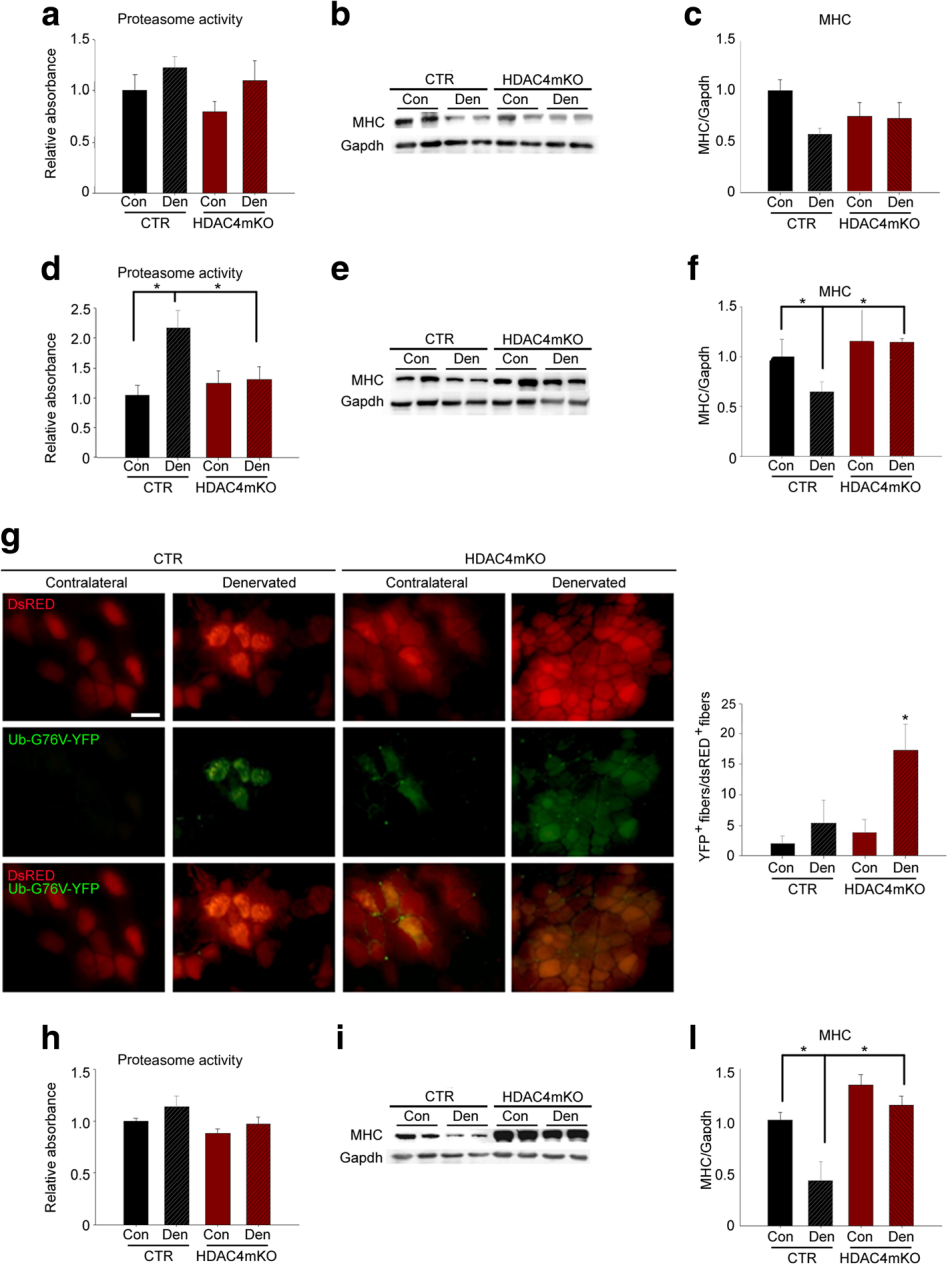
HDAC4 mediates the activation of ubiquitin-proteasome pathway in skeletal muscle upon denervation. **a** Proteasome activity in control (CTR) and HDAC4mKO muscles, 1 week following denervation. *n* = 3–4. **b** Representative western blot for MHC protein in control (CTR) and HDAC4mKO muscles, 1 week following denervation. Gapdh was used as loading control. **c** Densitometric measurements of MHC protein, 1 week following denervation. *n* = 4. **d** Proteasome activity in control (CTR) and HDAC4mKO muscles, 2 weeks following denervation. Data are shown as mean ± SEM; *n* = 3–4. Two-way ANOVA revealed an interaction (*F* = 5.48; df 1; *p* = 0.03); *p < 0.05 by Tukey’s HSD test. **e** Representative western blot for MHC protein in control (CTR) and HDAC4mKO muscles, 2 weeks following denervation. Gapdh was used as loading control. **f** Densitometric measurements of MHC protein, 2 weeks following denervation. *n* = 4; two-way ANOVA revealed an interaction (*F* = 4.96; df 1; *p* = 0.046); *p < 0.05 by Tukey’s HSD test. **g** Representative pictures of control (CTR) and HDAC4mKO muscles electroporated with Ub-G76 V-YFP (green), a UPS activity reporter and dsRED (red) plasmids, for monitoring the efficiency of electroporation, 2 weeks after denervation. Scale bar = 50 μm. Quantification of green and red positive fibers. *n* = 3; between 50 and 300 dsRED^+^ fibers were counted per sample. Two-way ANOVA revealed an interaction (*F* = 5.48; df 1; *p* = 0.03); *p < 0.05 by Tukey’s HSD test. **h** Proteasome activity in control (CTR) and HDAC4mKO muscles, 4 weeks following denervation. Data are shown as mean ± SEM; *n* = 3–4. **i** Representative western blot for MHC protein in control (CTR) and HDAC4mKO muscles, 4 weeks following denervation. Gapdh was used as loading control. **l** Densitometric measurements of MHC protein, 4 weeks following denervation. *n* = 4; two-way ANOVA revealed an interaction (*F* = 5.25; df 1; *p* = 0.04); *p < 0.05 by Tukey’s HSD test.

Skeletal muscle triggers UPS and autophagy to catabolize cellular components in response to denervation [4, 43, 44]. Since HDAC4 is a crucial mediator of skeletal muscle response to denervation, we monitored the activation of both UPS and autophagy in HDAC4mKO mice, over time. No significant differences were detected in the proteasome activity among samples, 1 week following denervation (Fig. 2a). Myosin heavy chain (MHC) levels were also monitored by western blot analyses (Fig. 2b), as an index of proteasome activity. Two-way ANOVA on MHC densitometry revealed that denervation significantly modulated MHC levels; however, no differences between genotypes occurred at this time point (Fig. 2c).

Conversely, 2 weeks following denervation, proteasome activity was significantly increased in denervated muscles of control mice, while it resulted not activated in HDAC4mKO muscles (Fig. 2d). Consistently, MHC levels significantly decreased in denervated control muscles, differently from HDAC4mKO mice, 2weeks following denervation (Fig. 2e, f). We further assessed proteasome activity by expressing a proteasome activity reporter plasmid, Ub-G76V-YFP, whose product is normally degraded by the proteasome and rapidly accumulates if proteasome activity is impaired [45]. dsRED plasmid was co-electroporated, in HDAC4mKO and control skeletal muscles, to monitor transfection efficiency. 3 days later, mice were subjected to denervation and after 2 weeks YFP^+^ and RED^+^ fibers were quantified. YFP fluorescence was sporadically observed in transfected control and HDAC4mKO contralateral fibers, but significantly increased in HDAC4mKO muscles following denervation (Fig. 2g), indicating an impairment of UPS activity.

Proteasome activity did not significantly differ among samples, 4 weeks after denervation (Fig. 2h), while differences in MHC levels were detected by western blot analyses (Fig. 2i). Indeed, denervated control muscles showed a significant decrease in MHC levels if compared to contralateral control muscles or denervated HDAC4mKO muscles (Fig. 2i, l). Overall, these data proved that HDAC4mKO were unable to activate the proteasome-ubiquitin pathway following denervation.

**Fig. 3:**
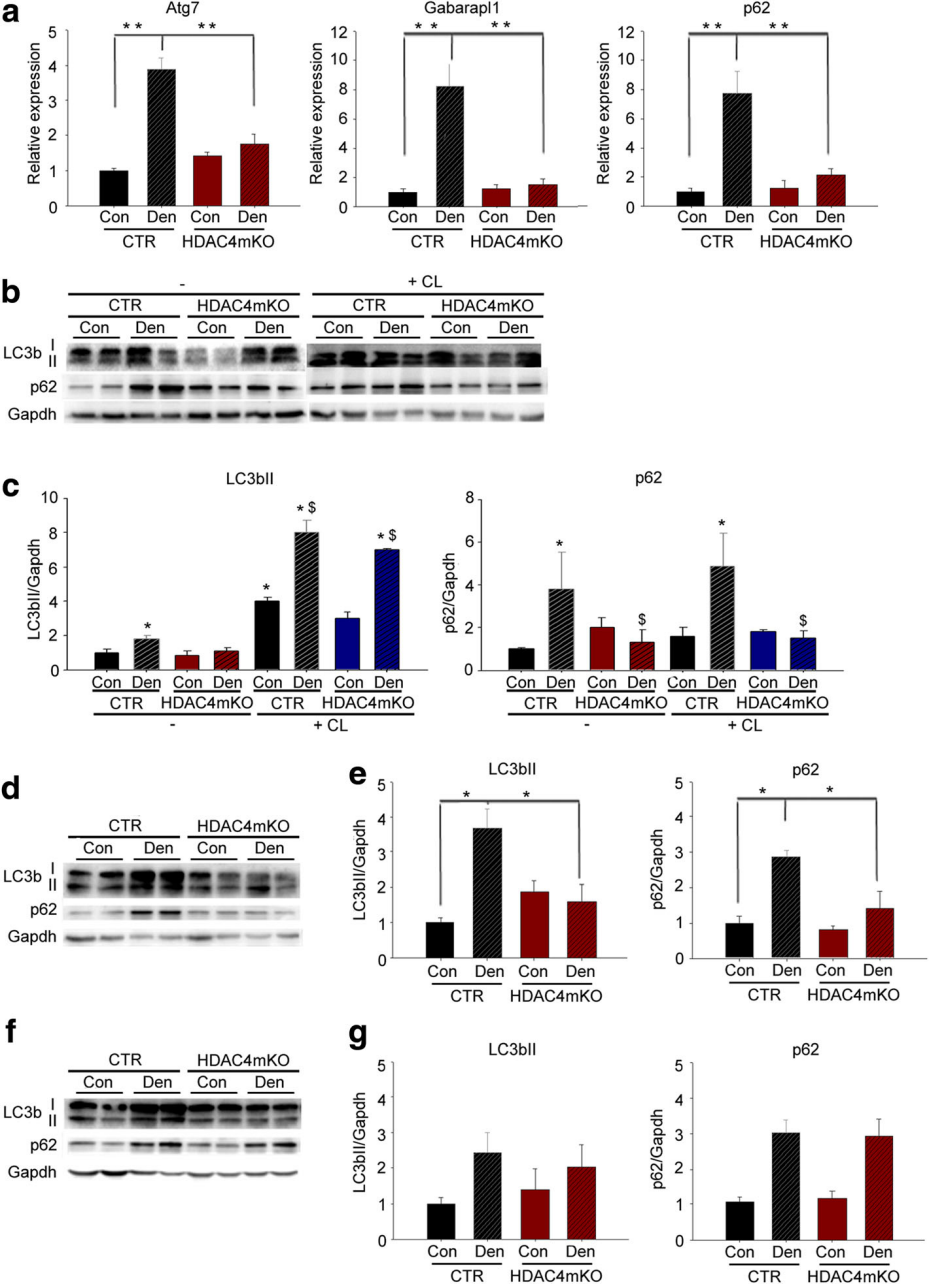
HDAC4 mediates the activation of autophagy in skeletal muscle upon denervation. **a** Real-time PCR for autophagic markers in control (CTR) and HDAC4mKO skeletal muscle, 1 week following denervation. Data are shown as mean ± SEM; *n* = 8; two-way ANOVA revealed an interaction (*F* = 29.3; df 1; *p* = 0.001 for Atg7; *F* = 28.5; df 1; *p* = 0.001 for Gabarapl1; *F* = 9.65; df 1; *p* = 0.004 for p62); **p < 0.01 by Tukey’s HSD test. **b** Representative western blot for LC3b and p62 proteins in control (CTR) and HDAC4mKO TA muscles, 1 week following denervation, in the absence (−) or after colchicine (CL) treatment. Gapdh was used as loading control. **c** Densitometric analyses for LC3b and p62 proteins, 1 week following denervation, in the absence (−) or after colchicine (CL) treatment. Data are shown as mean ± SEM; *n* = 3–4; two-way ANOVA revealed an interaction (*F* = 114; df 1; *p* = 0.001 for LC3b; *F* = 14.29; df 1; *p* = 0.036 for p62); *p < 0.05 versus CTR Con; $*p* < 0.05 versus CTR Den by Tukey’s HSD test. **d** Representative western blot for LC3b and p62 proteins, 2 weeks following denervation. Gapdh was used as loading control. **e** Densitometric measurements for LC3b and p62 proteins, 2 weeks following denervation. *n* = 3–4; two-way ANOVA revealed an interaction (*F* = 13.21; df 1; *p* = 0.046 for LC3b; *F* = 7.35; df 1; *p* = 0.01 for p62); *p < 0.05 versus CTR Con by Tukey’s HSD test. **f** Representative western blot analyses for LC3b and p62, 4 weeks after denervation. Gapdh was used as loading control. **g** Densitometric measurements for LC3b and p62 proteins, 4 weeks following denervation. *n* = 4; two-way ANOVA revealed an effect of denervation (*F* = 6.34; df 1; *p* = 0.028 for LC3b; *F* = 10.68; df 1; *p* = 0.0061 for p62)

To evaluate if HDAC4 mediates autophagy activation, we evaluated autophagy over time, following denervation. We first measured the expression of different autophagic markers in HDAC4mKO and control mice, 1 week after denervation. As expected, skeletal muscles of control mice strongly upregulated the expression of autophagic markers, i.e., *Atg7*, *Gabarapl1*, and *p62*, with respect to contralateral muscles (Fig. 3a). Interestingly, HDAC4mKO mice did not increase the expression of any of these markers following denervation (Fig. 3a). To confirm these findings, we analyzed LC3b and p62 protein levels (Fig. 3b). While denervation induced significant accumulation of the membrane-bound LC3bII and p62 in control mice, the induction was blunted in denervated HDAC4mKO muscles (Fig. 3b, c). A reduction in autophagic markers could depend on either an inhibition of the autophagic induction or an increase in the autophagic degradation of the cargo [10]. With the aim to discriminate between these two possibilities, we monitored the autophagic flux after administering colchicine (CL), which blocks the autophagosome-lysosome fusion [46]. CL treatment induced the accumulation of LC3bII in both contralateral and denervated muscles in control mice with respect to untreated ones, as expected (Fig. 3b, c), indicative of a block in the late stages of the autophagic flux. Similarly, CL treatment induced an accumulation of LC3bII in both contralateral and denervated HDAC4mKO muscles (Fig. 3b, c), indicative of a CL-mediated accumulation of autophagosomes in HDAC4mKO mice. However, differently from control mice, p62 protein was not accumulated after CL treatment in HDAC4mKO mice (Fig. 3b, c). Taken together, the autophagic flux experiment indicated that HDAC4mKO mice could form autophagosomes in response to denervation but displayed a problem in the autophagic flux between the autophagosome formation and the autophagosome-lysosome fusion.

Autophagy was further analyzed 2 and 4 weeks following denervation, by western blot analyses for LC3b and p62. After 2 weeks of denervation we found that, similarly to 1 week, denervation significantly increased LC3bII and p62 protein levels only in control mice (Fig. 3d, e). After 4 weeks, instead, denervation significantly increased LC3bII and p62 levels but no differences were registered between genotypes (Fig. 3f, g).

### Methylene blue and intermittent fasting ameliorate HDAC4mKO muscle following denervation

To evaluate the relative contribution of UPS and of the autophagic activation to the loss of muscle integrity and inflammation in HDAC4mKO mice following denervation, we administered either methylene blue (MB) to activate the proteasome [42], or intermittent fasting (IF) to triggered autophagy [47]. We first confirmed that MB activated UPS in HDAC4mKO mice by measuring proteasome activity (Additional file 1: Figure S3a). To further demonstrate that MB effectively activated the proteasome in HDAC4mKO mice, we evaluated MHC levels by western blot analyses. MB treatment resulted in lower MHC levels in HDAC4mKO mice, whereas denervation did not affect MHC levels in untreated HDAC4mKO mice (Additional file 1: Figure S3b). Moreover, the proteasome reporter activity plasmid, Ub-G76 V-YFP, was transfected with dsRED in HDAC4mKO mice, and YFP^+^ and RED^+^ fibers were counted 2 weeks following denervation, without or with MB treatment (Additional file 1: Figure S3c). Quantification of YFP^+^ and RED^+^ fibers proved that MB treatment efficiently reduced the YFP^+^ fibers accumulated in HDAC4mKO upon denervation (Additional file 1: Figure S3c). We also proved that IF significantly upregulated gene expression and protein levels of autophagic markers in HDAC4mKO mice (Additional file 1: Figure S3d and e).

**Fig. 4:**
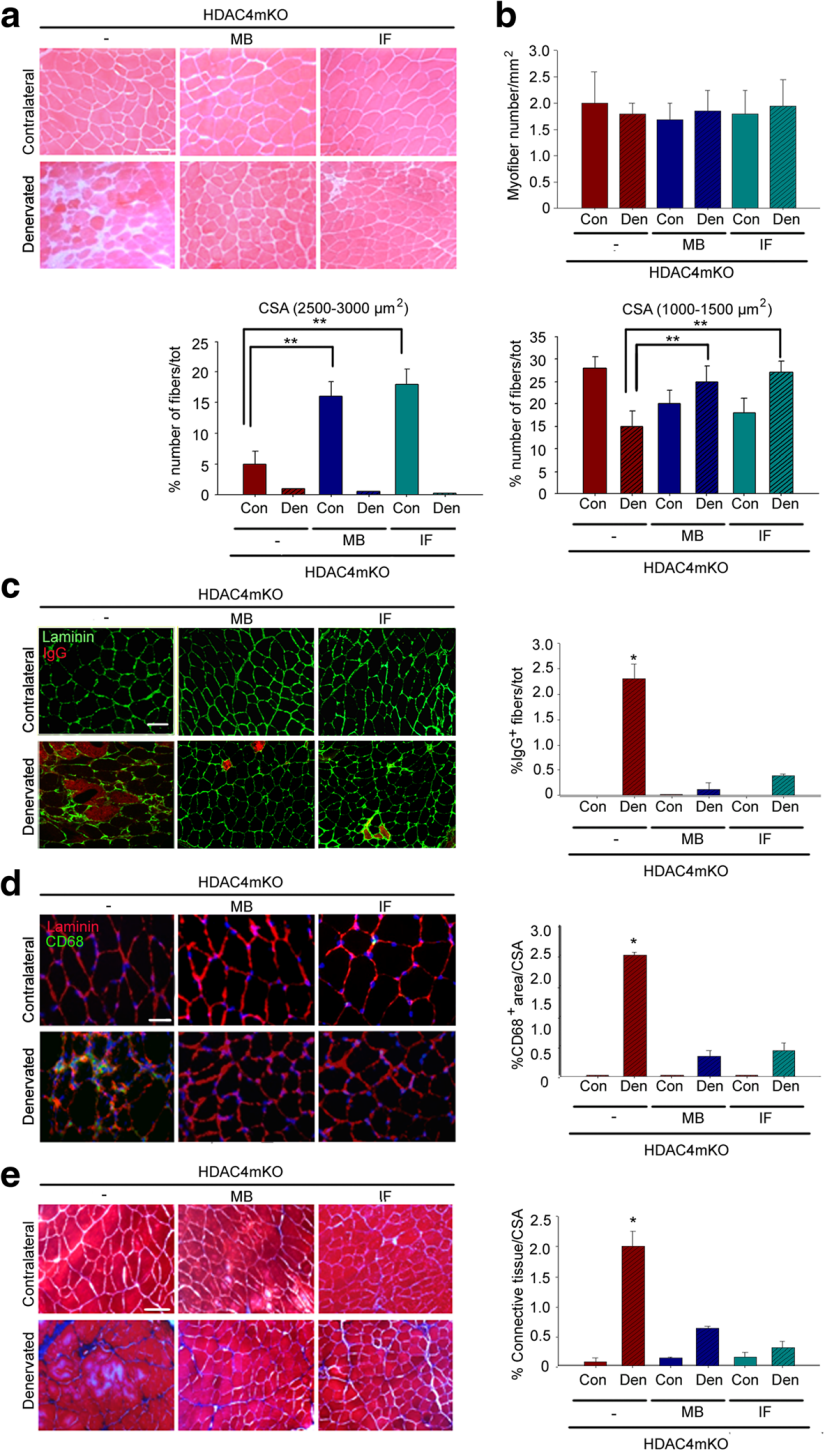
Methylene blue and intermittent fasting preserve HDAC4mKO skeletal muscle structure following denervation. **a** Hematoxylin and eosin staining of HDAC4mKO contralateral and denervated muscles, 4 weeks after denervation, without (−) or after MB or IF treatment. Scale bar = 50 μm. **b** Number of myofibers in HDAC4mKO contralateral and denervated muscles, 4 weeks after denervation, without (−) or after MB or IF treatment. *n* = 3. Number of HDAC4mKO myofibers with 2500–3000 or 1000–1500 μm^2^ cross-sectional area, without (−) or after MB or IF treatment, 4 weeks after denervation. Data are shown as mean ± SEM; *n* = 4; two-way ANOVA revealed an interaction (*F* = 6.72; df 1; *p* = 0.02 for MB; *F* = 8.2; df 1; *p* = 0.01 for IF) (*F* = 7.27; df 1; *p* = 0.026 for MB; *F* = 10.2; df 1; *p* = 0.01 for IF); **p < 0.01 by Tukey’s HSD test. **c** HDAC4mKO muscle sections labeled with laminin (green) and IgG (red), 4 weeks after denervation, without (−) or after MB or IF treatments, with relative quantification. Scale bar = 50 μm. *n* = 3; two-way ANOVA showed an interaction (*F* = 10.82; df 1; *p* = 0.0094 for MB; *F* = 11.43; df 1; *p* = 0.0081 for IF); *p < 0.05 by Tukey’s HSD test. **d** Immunostaining for CD68 (green) and laminin (red) of HDAC4mKO muscles 4 weeks after denervation, without (−) or after MB or IF treatment, with relative quantification**.** Scale bar = 25 μm. *n* = 3; two-way ANOVA showed an interaction (*F* = 6.57; df 1; *p* = 0.03 for MB; *F* = 6.06; df 1; *p* = 0.036 for IF); *p < 0.05 by Tukey’s HSD test. **e** Masson’s trichrome staining of HDAC4mKO contralateral and denervated muscles, 4 weeks after denervation, without (−) or after MB or IF treatment, with relative quantification. Scale bar = 50 μm. *n* = 3; two-way ANOVA showed an interaction (*F* = 53.95; df 1; *p* = 0.001 for MB and *F* = 59.36; df 1; *p* = 0.0001 for IF); *p < 0.05 by Tukey’s HSD test.

In order to assess the bearing of forced activation of the catabolic pathways upon the loss of muscle integrity and inflammation, HDAC4mKO mice were subjected to either MB treatment or IF since the day of surgery, and histological and morphometric analyses were performed 4 weeks following denervation. Strikingly, HDAC4mKO muscles treated with either MB or IF displayed a better preserved structural integrity, following denervation (Fig. 4a). No significant differences in the number of myofibers were registered among samples; while, analysis of myofiber CSA distribution revealed that both MB- and IF-treated HDAC4mKO mice displayed a significant higher number of larger myofibers (2500–3000 μm²) in contralateral muscles and an increase in the number of smaller myofibers (1000–1500 μm²) following denervation, with respect to untreated HDAC4mKO mice (Fig. 4b and Additional file 1: Figure S4a and b). However, no effects were registered on muscle mass following treatments (Additional file 1: Figure S4c). Furthermore, muscle integrity, macrophagic infiltration and amount of connective tissue were evaluated. Quantification of IgG^+^ fibers, CD68^+^, or Masson’s trichrome^+^ area showed that both MB and IF treatments reduced necrotic fibers, macrophagic infiltration, and connective tissue in denervated HDAC4mKO muscles (Fig. 4c–e).

### HDAC4 mediates the activation of OS response in skeletal muscle upon denervation

**Fig. 5:**
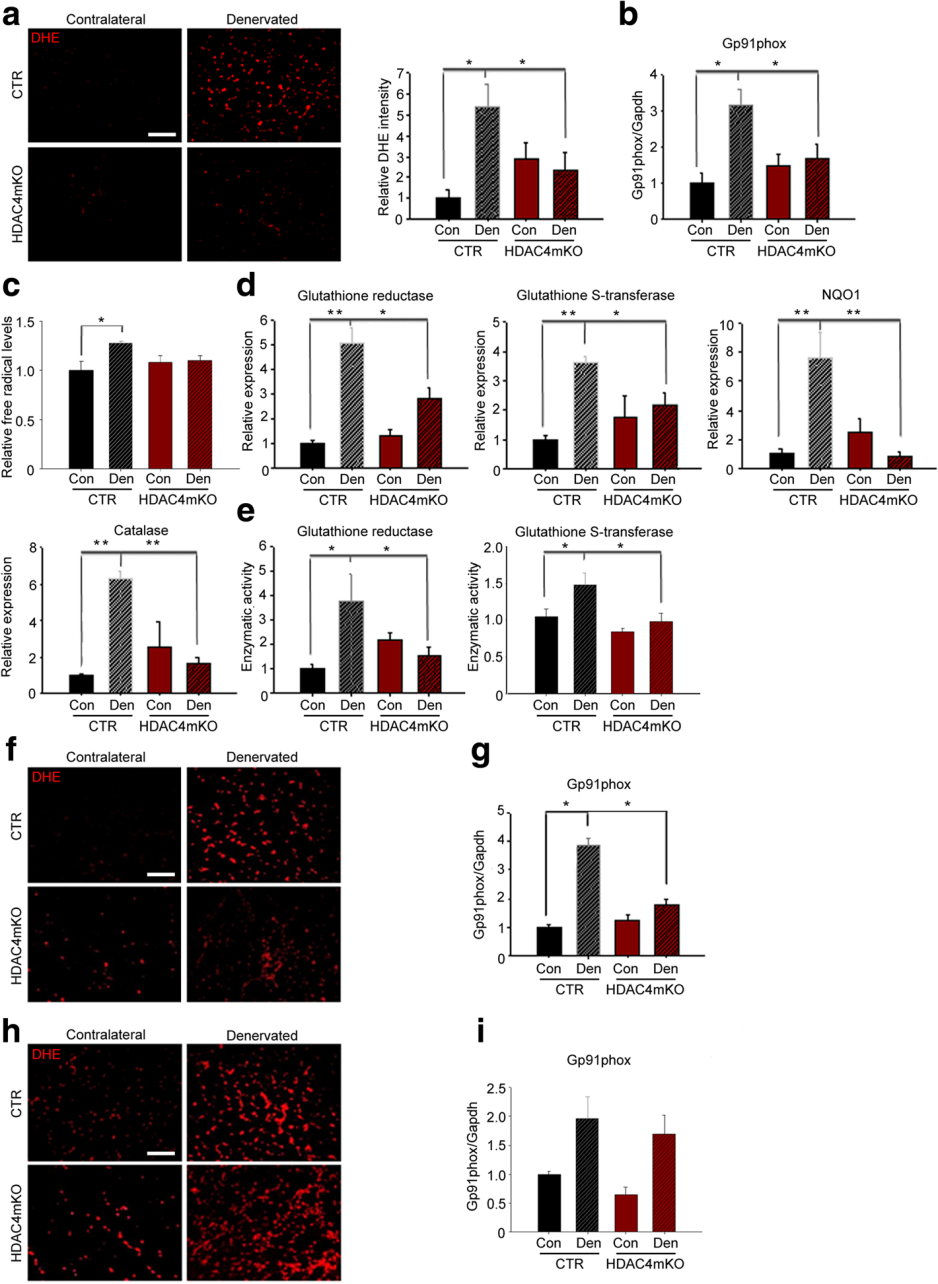
HDAC4 mediates the activation of OS response in skeletal muscle upon denervation. **a** DHE staining of control (CTR) and HDAC4mKO TA muscles following 1 week of denervation and relative quantification. Scale bar = 50 μm. *n* = 5; two-way ANOVA showed an interaction (*F* = 8.74; df 1; *p* = 0.01); *p < 0.05 by Tukey’s HSD test. **b** Densitometric analysis of western blots for Gp91phox in CTR and HDAC4mKO TA muscles, 1 week after denervation. *n* = 4; two-way ANOVA showed an interaction (*F* = 20.14; df 1; *p* = 0.0004); *p < 0.05 by Tukey’s HSD test. **c** Quantification of ROS and RNS 1 week of denervation. *n* = 4–5; two-way ANOVA revealed an effect of denervation (*F* = 9; df 1; *p* = 0.009) and an interaction (*F* = 5; df 1; *p* = 0.041); *p < 0.05 by Tukey’s HSD test. **d** Real-time PCR of antioxidant enzymes in CTR and HDAC4mKO skeletal muscles, 1 week following denervation. *n* = 4–5; two-way ANOVA revealed an interaction (*F* = 8.82; df 1; *p* = 0.0082 for GSR; *F* = 5.24; df 1; *p* = 0.03 for GST; *F* = 14.07; df 1; *p* = 0.0015 for NQO1 and *F* = 6.14; df 1; *p* = 0.02 for catalase); *p < 0.05; **p < 0.01 by Tukey’s HSD test. **e** Activity of GSR and GST, 1 week following denervation. *n* = 5–6; two-way ANOVA showed an interaction (*F* = 11.16; df 1; *p* = 0.0026 for GSR; *F* = 6.16; df 1; *p* = 0.026 for GST); *p < 0.05 by Tukey’s HSD test. **f** DHE staining of CTR and HDAC4mKO TA muscles following 2 weeks of denervation. Scale bar = 50 μm. **g** Densitometric analysis of western blots for Gp91phox in CTR and HDAC4mKO TA muscles, 2 weeks after denervation. *n* = 4; two-way ANOVA showed an interaction (*F* = 36.77; df 1; *p* = 0.0001); *p < 0.05 by Tukey’s HSD test. **h** DHE staining of CTR and HDAC4mKO TA muscles following 4 weeks of denervation. Scale bar = 50 μm. **i** Densitometric analysis of western blots for Gp91phox in CTR and HDAC4mKO TA muscles, 4 weeks after denervation. *n* = 4; two-way ANOVA revealed an effect of denervation (*F* = 14.97; df 1; *p* = 0.0026)

Since HDAC4 modulates skeletal muscle response following denervation, which increases the levels of OS, we wondered whether HDAC4 mediates the response to OS. Oxidative stress response and oxidative stress levels were evaluated over time. Reactive oxygen species (ROS) levels were assessed and quantified by dihydroethidium (DHE) staining, at 1, 2, and 4 weeks following denervation. Unlike control mice, which showed significant upregulation of DHE intensity, HDAC4mKO mice did not display increased levels of ROS 1 week following denervation (Fig. 5a). This result was corroborated by the evaluation of Gp91phox, a marker of OS. Indeed, densitometric analysis of Gp91phox levels showed a significant increase in denervated control muscles, if compared to contralateral ones. Conversely, in HDAC4mKO mice, the increase of Gp91phox levels 1 week following denervation was significantly blunted (Fig. 5b and Additional file 1: Figure S5a). Oxidative stress was also quantified from muscle extracts by measuring free radical levels. Denervation significantly increased ROS and reactive nitrogen species (RNS) in control muscles, but not in HDAC4mKO mice, 1 week following denervation (Fig. 5c). Moreover, we quantified gene expression of several genes involved in the oxidative stress response, i.e., glutathione reductase (GSR), glutathione S-transferase (GST), NAD(P)H dehydrogenase [quinone] 1 (NQO1), and catalase (Fig. 5d). For these markers, the expression resulted significantly upregulated following denervation in controls but not in HDAC4mKO muscles (Fig. 5d). The enzymatic activity of glutathione reductase and glutathione S-transferase showed the same modulation as the expression levels (Fig. 5e), confirming that control mice significantly upregulated the OS response following denervation, while HDAC4mKO mice did not increase either the expression or the enzymatic activity of genes involved in the OS response.

Oxidative stress was further analyzed after 2 and 4 weeks of denervation. After 2 weeks, we observed that denervation increased OS levels only in control mice (Fig. 5f, g and Additional file 1: Figure S5b), similarly to 1 week following denervation. After 4 weeks, although denervation resulted in a significant increase of OS in both control and HDAC4mKO mice (Fig. 5h, i and Additional file 1: Figure S5c), gene expression of GSR, GST, NQO1, and catalase resulted not significantly modulated in denervated HDAC4mKO muscles (Additional file 1: Figure S5d). These data point to the importance of HDAC4 in activating the OS response following denervation in skeletal muscle.

### Administration of an antioxidant drug prevents the loss of structural integrity in HDAC4mKO muscles following denervation

**Fig. 6:**
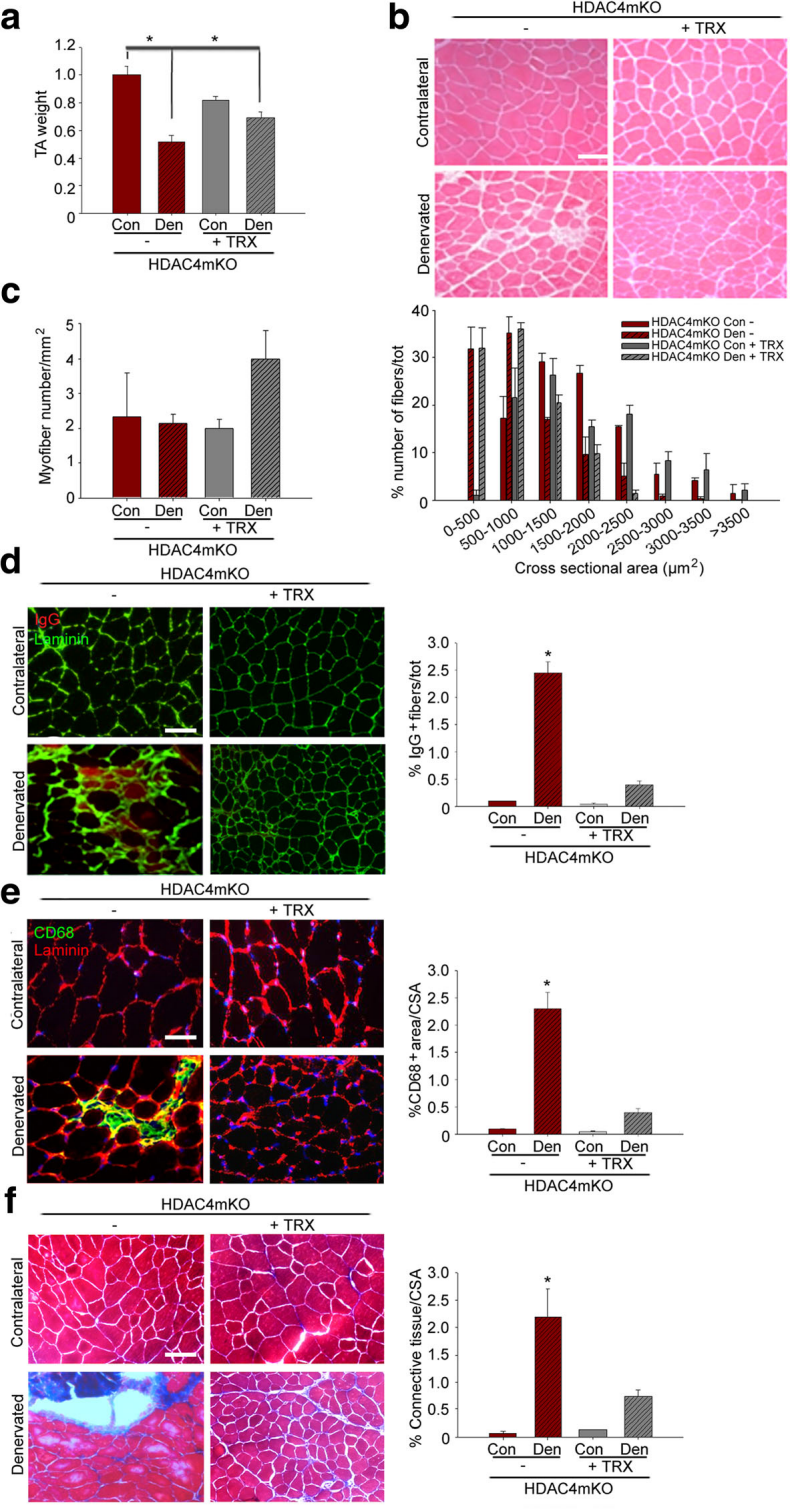
Administration of an antioxidant drug ameliorates HDAC4mKO muscle structure following denervation. **a** Weight of HDAC4mKO TA muscles without (−) or with Trolox (TRX) treatment, over contralateral ones, 4 weeks after denervation. Data are shown as mean ± SEM; *n* = 3; two-way ANOVA revealed an interaction (*F* = 12; df 1; *p* = 0.0085); *p < 0.05 by Tukey’s HSD test. **b** Hematoxylin and eosin staining of HDAC4mKO contralateral and denervated muscles, 4 weeks after denervation, without (−) or with Trolox treatment. Scale bar = 50 μm. **c** Number of myofibers in HDAC4mKO contralateral and denervated muscles, 4 weeks after denervation, without (−) or after Trolox treatment. *n* = 3. Quantitative distribution analysis of fiber cross-sectional area of HDAC4mKO mice, following 4 weeks of denervation, without or with Trolox treatment. Data are shown as mean ± SEM. *n* = 3; two-way ANOVA revealed an effect of denervation (*F* = 7.25; df 1; *p* = 0.021 for 500–1000 μm^2^ class). **d** HDAC4mKO muscles labeled for laminin (green) and IgG (red), 4 weeks after denervation and relative quantification. Scale bar = 50 μm. *n* = 3; two-way ANOVA revealed an interaction (*F* = 9.26; df 1; *p* = 0.013); *p < 0.05 by Tukey’s HSD test. **e** Immunostaining for CD68 (green) and laminin (red) of HDAC4mKO muscles, 4 weeks after denervation, without (−) or with Trolox treatment, with relative quantification. Scale bar = 25 μm. *n* = 3; two-way ANOVA revealed an interaction (*F* = 6.07; df 1; *p* = 0.035); *p < 0.05 by Tukey’s HSD test. **f** Masson’s trichrome staining of HDAC4mKO contralateral and denervated muscles, 4 weeks after denervation, without (−) or with Trolox treatment, with relative quantification. Scale bar = 50 μm. *n* = 3; two-way ANOVA revealed an interaction (*F* = 38.75; df 1; *p* = 0.0003); *p < 0.05 by Tukey’s HSD test.

Since HDAC4mKO mice did not activate OS response upon denervation, to evaluate if the impairment contributed to HDAC4mKO skeletal muscle integrity following denervation, we treated HDAC4mKO mice with Trolox, a cell-permeable derivative of vitamin E with antioxidant properties [24, 48, 49], for 4 weeks, after the surgery. Administration of Trolox efficiently reduced ROS levels in HDAC4mKO mice following denervation (Additional file 1: Figure S6a and b). Strikingly, Trolox treatment significantly increased muscle mass of HDAC4mKO mice following denervation (Fig. 6a). Histological analyses showed that HDAC4mKO muscle architecture was more preserved upon denervation following Trolox treatment (Fig. 6b). Morphometric analyses demonstrated that Trolox neither significantly affected the number of myofibers in HDAC4mKO mice nor myofiber distribution (Fig. 5c), retaining the resistance to neurogenic muscle atrophy. IgG and immunostaining analyses revealed that Trolox significantly reduced the number of necrotic fibers (Fig. 6d), macrophagic infiltration (Fig. 6e), and connective tissue (Fig. 6f) in denervated HDAC4mKO muscles with respect to untreated once.

## Discussion

HDAC4 is a crucial mediator of skeletal muscle response to denervation, contributing to the induction of the expression of the muscle-specific E3 ubiquitin-ligases and miR-206, and activating the MAPK-AP1 axis [31, 35]. Numerous pathologies that affect the peripheral nervous system, causing the loss or the alteration of nervous stimuli, lead to decreased skeletal muscle mass and functionality. Even in sarcopenia, the age-related loss of skeletal muscle mass, a strong association between motoneuron impairment and the degree of muscle wasting has been established. Indeed, numerous studies showed that degeneration and loss of motor neurons followed by structural and functional changes in innervations, significantly contribute to the progression of sarcopenia [50–52]. Considering the central role in mediating skeletal muscle nerve response, several studies indicated HDAC4 as a potential therapeutic target for the prevention of neurogenic muscle atrophy in pathological conditions or during aging [38, 53]. In all these conditions, the proposed treatments should be prolonged for months or years. In the present work, we proved that inhibition of HDAC4 in long-term denervation condition is deleterious for skeletal muscle. Contrary to controls, HDAC4mKO mice showed loss of skeletal muscle integrity, characterized by the presence of necrotic fibers, macrophage infiltration, accumulation of connective tissue, and heterogeneity in myofiber size following 4 weeks of denervation. However, no significant differences in myofiber number or muscle weights were registered between HDAC4mKO and control mice, thus suggesting that loss of muscle mass in HDAC4mKO mice depends on loss of muscle integrity and not merely on muscle atrophy.

To investigate the molecular mechanisms underlying this phenotype, we evaluated the activation of the catabolic pathways triggered in neurogenic muscle atrophy, i.e., the UPS and autophagy. Indeed, if UPS or autophagic hyper-activation leads to muscle atrophy [54, 55], their impairment in conditions in which they must be activated is deleterious for skeletal muscle [56, 57]. While control mice activated the proteasome and degraded MHC upon denervation, HDAC4mKO mice did not show either proteasome activation or decrease in MHC levels, until 4 weeks following denervation, indicating an impairment in the ubiquitin-proteasome system response. Autophagic flux experiments demonstrated that autophagosome formation occurred in HDAC4mKO muscles, 1 week following denervation. However, accumulation of autophagic intermediates failed in HDAC4mKO upon denervation, after 1 and 2 weeks. After 4 weeks, instead, denervation significantly increased LC3bII and p62 both in control and HDAC4mKO mice, indicating that autophagy was induced also in muscles with HDAC4 deletion.

Although proteasome inhibitors can hinder muscle atrophy in different animal models [58–60], long-term inhibition of this pathway could be deleterious for muscle cells. Indeed, the administration of Bortezomib, a proteasome inhibitor, FDA-approved for the treatment of multiple myeloma, causes cardiac complications in chronic patients [61]. This observation is in accordance with our results, which showed that long-term inhibition of the UPS, upon denervation, induced loss of muscle integrity, similarly to mice null for MuRF1 [55].

As for UPS, autophagy plays a major role in removing damaged mitochondria and in mediating the metabolic response of skeletal muscle to denervation, without inducing neurogenic muscle atrophy [24]. A proper autophagic response or flux is crucial for the maintenance of skeletal muscle homeostasis, both in physiological and in pathological conditions [41, 62, 63]. By triggering either UPS or autophagy, loss of membrane integrity, inflammatory infiltration, and accumulation of fibrotic tissue were efficiently rescued in HDAC4mKO mice upon long-term denervation. Moreover, morphometric measurements of myofiber cross-sectional area confirmed that the treatments induced a change in denervated and contralateral HDAC4mKO muscle fiber size, restoring a myofiber distribution similar to that of control mice.

OS plays a dualistic role in skeletal muscle: on the one hand, low levels of OS contribute to the maintenance of muscle homeostasis in different physiological conditions, e.g., physical activity [64–66]; on the other hand, excessive ROS production leads to alteration of skeletal muscle homeostasis and is involved in muscle damage in several pathological conditions [67–69]. Since neurodegenerative conditions and aging have been associated with chronically elevated levels of ROS, antioxidant treatments have been proposed or are currently in clinical trial [70–72]. In our work, we observed that HDAC4mKO mice neither increased the levels of ROS and RNS in skeletal muscle nor induced the expression and activation of the enzymes involved in the antioxidant response, 1 week following denervation. 2 weeks following denervation, HDAC4mKO mice still showed decreased levels of OS, compared to control mice. After 4 weeks, although no significant differences in OS levels were registered between the genotypes, HDAC4mKO mice still showed compromised activation of the expression of genes involved in the OS response. Therefore, we speculate that the changes in OS levels observed following 4 weeks of denervation are a consequence of loss of muscle integrity. Importantly, antioxidant treatment, not only prevented loss of muscle integrity in HDAC4mKO mice, with a reduction of necrotic fibers, macrophagic infiltration, and connective tissue but also preserved muscle mass in denervated muscles. In other words, antioxidant treatment preserved all the positive effects of HDAC4 deletion in skeletal muscle following denervation, i.e., the resistance to neurogenic muscle atrophy, abrogating the negative consequences of a prolonged inhibition of the catabolic pathways, i.e., loss of structural integrity. Our data are in apparent contrast with the study in which administration of a pan-HDAC inhibitor prevents muscle atrophy following denervation, by increasing OS response and reducing oxidative damage [54]. However, the experimental differences between the two studies must be considered: (1) pan-HDAC inhibitors not specifically inhibit all members of HDAC superfamily; (2) systemic administration of HDAC inhibitors affects all tissues, differently from studying the effects on a tissue-specific KO mouse; (3) administration of HDAC inhibitors starting 3 weeks before denervation may provide different results from the deletion of HDAC4 since the embryonic stage E8.5, as in HDAC4mKO mice. Of note, independent groups showed a toxic effect of the Cre recombinase expression in different murine tissues [73–77]. Although a toxic effect of the myogenin;Cre mouse line was not reported, we cannot rule out the possibility that some of the effects in HDAC4mKO mice depended on Cre expression.

## Conclusion

In conclusion, our results reveal a novel role of HDAC4 in mediating the activation of OS response, UPS, and autophagy in skeletal muscle following denervation. We clearly showed that inhibition of HDAC4 could be deleterious for skeletal muscle in long-term denervation conditions, such as aging or neuromuscular disorders. These findings are relevant considering potential therapies for neurogenic muscle atrophy based on HDAC inhibitors. Indeed, HDAC inhibitors have been strongly recommended for the treatment of neurodegenerative diseases. However, neither the molecular mechanisms underlying their actions nor the possible long-term collateral effects have been clarified yet. Treatment with HDAC inhibitors may indirectly affect the acetylation of HDAC4 targets since HDAC4 does not possess direct histone deacetylase activity but acts through class I HDACs. By delineating new functions of HDAC4 in neurogenic muscle atrophy, our work represents a step-forward toward the development of efficient pharmaceutical approaches for neurogenic muscle atrophy, possibly by combining HDAC inhibitors with antioxidant drugs or with other treatments aiming to boost the catabolic pathways in skeletal muscle, otherwise compromised using HDAC inhibitors.

## Additional file

Additional file 1: **Figure S1.** Denervation differentially affected HDAC4mKO and control mice. **Figure S2.** HDAC4mKO muscles did not show differences in dystophin glycoprotein complex. **Figure S3.** Methylene blue and intermittent fasting efficiently activated UPS and autophagy in HDAC4mKO mice, respectively. **Figure S4.** Effects of methylene blue and intermittent fasting on HDAC4mKO muscles. **Figure S5.** HDAC4mKO mice showed altered levels of Gp91phox upon denervation. **Figure S6.** Trolox treatment efficiently reduces free radical levels in HDAC4mKO mice. (DOC 3765 kb)

## Abbreviations

ALS: Amyotrophic lateral sclerosis
Atg: Autophagy-related genes
Atg7: Autophagy-related gene 7
CL: Colchicine
CSA: Cross-sectional area
DHE: Dihydroethidium
DMSO: Dimethyl sulfoxide
GSR: Glutathione reductase
GST: Glutathione S-transferase
HDAC1: Histone deacetylase 1
HDAC2: Histone deacetylase 2
HDAC4: Histone deacetylase 4
HDAC6: Histone deacetylase 6
IF: Intermittent fasting
LC3b: Microtubule-associated proteins 1A/1B light chain 3B
MAPK-AP1: Mitogen-activated protein *kinases*-AP1
MB: Methylene blue
MHC: Myosin heavy chain
NQO1: NAD(P)H dehydrogenase [quinone] 1
OS: Oxidative stress
PINK1: PTEN-induced putative kinase 1
RNS: Reactive nitrogen species
ROS: Reactive oxygen species
SMA: Spinal muscular atrophy
TRX: Trolox
UPS: Ubiquitin proteasome system

## References

[1] Eley HL, Tisdale MJ (2007). Skeletal muscle atrophy, a link between depression of protein synthesis and increase in degradation. J Biol Chem.

[2] Glass DJ (2003). Molecular mechanisms modulating muscle mass. Trends Mol Med.

[3] Sandri M, Sandri C, Gilbert A, Skurk C, Calabria E, Picard A (2004). Foxo transcription factors induce the atrophy-related ubiquitin ligase atrogin-1 and cause skeletal muscle atrophy. Cell.

[4] Bodine SC, Latres E, Baumhueter S, Lai VK, Nunez L, Clarke BA (2001). Identification of ubiquitin ligases required for skeletal muscle atrophy. Science.

[5] O'Leary MF, Hood DA (2009). Denervation-induced oxidative stress and autophagy signaling in muscle. Autophagy.

[6] O'Leary MF, Vainshtein A, Carter HN, Zhang Y, Hood DA (2012). Denervation-induced mitochondrial dysfunction and autophagy in skeletal muscle of apoptosis-deficient animals. Am J Physiol Cell Physiol.

[7] Le Grand JN, Chakrama FZ, Seguin-Py S, Fraichard A (2011). age-Mourroux R, Jouvenot M, et al. GABARAPL1 (GEC1): original or copycat?. Autophagy.

[8] Seibenhener ML, Geetha T, Wooten MW (2007). Sequestosome 1/p62--more than just a scaffold. FEBS Lett.

[9] Shvets E, Fass E, Scherz-Shouval R, Elazar Z (2008). The N-terminus and Phe52 residue of LC3 recruit p62/SQSTM1 into autophagosomes. J Cell Sci.

[10] Klionsky DJ, Abdelmohsen K, Abe A, Abedin MJ, Abeliovich H, Acevedo AA (2016). Guidelines for the use and interpretation of assays for monitoring autophagy (3rd edition). Autophagy.

[11] Kane LA, Lazarou M, Fogel AI, Li Y, Yamano K, Sarraf SA (2014). PINK1 phosphorylates ubiquitin to activate Parkin E3 ubiquitin ligase activity. J Cell Biol.

[12] Shaw GS (2014). Switching on ubiquitylation by phosphorylating a ubiquitous activator. Biochem J.

[13] Narendra D, Tanaka A, Suen DF, Youle RJ (2008). Parkin is recruited selectively to impaired mitochondria and promotes their autophagy. J Cell Biol.

[14] Narendra DP, Jin SM, Tanaka A, Suen DF, Gautier CA, Shen J (2010). PINK1 is selectively stabilized on impaired mitochondria to activate Parkin. PLoS Biol.

[15] Cuervo AM, Stefanis L, Fredenburg R, Lansbury PT, Sulzer D (2004). Impaired degradation of mutant alpha-synuclein by chaperone-mediated autophagy. Science.

[16] Ding WX, Yin XM (2008). Sorting, recognition and activation of the misfolded protein degradation pathways through macroautophagy and the proteasome. Autophagy.

[17] Johnston JA, Ward CL, Kopito RR (1998). Aggresomes: a cellular response to misfolded proteins. J Cell Biol.

[18] Webb JL, Ravikumar B, Atkins J, Skepper JN, Rubinsztein DC (2003). Alpha-Synuclein is degraded by both autophagy and the proteasome. J Biol Chem.

[19] Kirkin V, Lamark T, Sou YS, Bjorkoy G, Nunn JL, Bruun JA (2009). A role for NBR1 in autophagosomal degradation of ubiquitinated substrates. Mol Cell.

[20] Milani M, Rzymski T, Mellor HR, Pike L, Bottini A, Generali D (2009). The role of ATF4 stabilization and autophagy in resistance of breast cancer cells treated with Bortezomib. Cancer Res.

[21] Pankiv S, Clausen TH, Lamark T, Brech A, Bruun JA, Outzen H (2007). p62/SQSTM1 binds directly to Atg8/LC3 to facilitate degradation of ubiquitinated protein aggregates by autophagy. J Biol Chem.

[22] Wang XJ, Yu J, Wong SH, Cheng AS, Chan FK, Ng SS (2013). A novel crosstalk between two major protein degradation systems: regulation of proteasomal activity by autophagy. Autophagy.

[23] Wu WK, Cho CH, Lee CW, Wu YC, Yu L, Li ZJ (2010). Macroautophagy and ERK phosphorylation counteract the antiproliferative effect of proteasome inhibitor in gastric cancer cells. Autophagy.

[24] Pigna E, Greco E, Morozzi G, Grottelli S, Rotini A, Minelli A (2017). Denervation does not induce muscle atrophy through oxidative stress. Eur J Transl Myol.

[25] Sandri M (2010). Autophagy in skeletal muscle. FEBS Lett.

[26] Shang F, Taylor A (2011). Ubiquitin-proteasome pathway and cellular responses to oxidative stress. Free Radic Biol Med.

[27] Lunke S, El-Osta A (2009). The emerging role of epigenetic modifications and chromatin remodeling in spinal muscular atrophy. J Neurochem.

[28] Tajrishi MM, Shin J, Hetman M, Kumar A (2014). DNA methyltransferase 3a and mitogen-activated protein kinase signaling regulate the expression of fibroblast growth factor-inducible 14 (Fn14) during denervation-induced skeletal muscle atrophy. J Biol Chem.

[29] Moresi V, Marroncelli N, Pigna E, Sergio A, Tollefsboll TO (2016). Epigenetic of muscle disorders. Medical Epigenetics.

[30] Choi MC, Cohen TJ, Barrientos T, Wang B, Li M, Simmons BJ (2012). A direct HDAC4-MAP kinase crosstalk activates muscle atrophy program. Mol Cell.

[31] Moresi V, Williams AH, Meadows E, Flynn JM, Potthoff MJ, McAnally J (2010). Myogenin and class II HDACs control neurogenic muscle atrophy by inducing E3 ubiquitin ligases. Cell.

[32] Lee JY, Koga H, Kawaguchi Y, Tang W, Wong E, Gao YS (2010). HDAC6 controls autophagosome maturation essential for ubiquitin-selective quality-control autophagy. EMBO J.

[33] Moresi V, Carrer M, Grueter CE, Shelton JM, Richardson JA, Rifki OF (2012). Histone deacetylases 1 and 2 regulate autophagy flux and skeletal muscle homeostasis in mice. Proc Natl Acad Sci U S A.

[34] Hariharan N, Maejima Y, Nakae J, Paik J, Depinho RA, Sadoshima J (2010). Deacetylation of FoxO by Sirt1 plays an essential role in mediating starvation-induced autophagy in cardiac myocytes. Circ Res.

[35] Cohen TJ, Waddell DS, Barrientos T, Lu Z, Feng G, Cox GA (2007). The histone deacetylase HDAC4 connects neural activity to muscle transcriptional reprogramming. J Biol Chem.

[36] Baehr LM, West DW, Marcotte G, Marshall AG, De Sousa LG, Baar K (2016). Age-related deficits in skeletal muscle recovery following disuse are associated with neuromuscular junction instability and ER stress, not impaired protein synthesis. Aging (Albany NY).

[37] Bricceno KV, Sampognaro PJ, Van Meerbeke JP, Sumner CJ, Fischbeck KH, Burnett BG (2012). Histone deacetylase inhibition suppresses myogenin-dependent atrogene activation in spinal muscular atrophy mice. Hum Mol Genet.

[38] Bruneteau G, Simonet T, Bauche S, Mandjee N, Malfatti E, Girard E (2013). Muscle histone deacetylase 4 upregulation in amyotrophic lateral sclerosis: potential role in reinnervation ability and disease progression. Brain.

[39] Walsh ME, Bhattacharya A, Sataranatarajan K, Qaisar R, Sloane L, Rahman MM (2015). The histone deacetylase inhibitor butyrate improves metabolism and reduces muscle atrophy during aging. Aging Cell.

[40] Potthoff MJ, Wu H, Arnold MA, Shelton JM, Backs J, McAnally J (2007). Histone deacetylase degradation and MEF2 activation promote the formation of slow-twitch myofibers. J Clin Invest.

[41] Raben N, Hill V, Shea L, Takikita S, Baum R, Mizushima N (2008). Suppression of autophagy in skeletal muscle uncovers the accumulation of ubiquitinated proteins and their potential role in muscle damage in Pompe disease. Hum Mol Genet.

[42] Medina DX, Caccamo A, Oddo S (2011). Methylene blue reduces abeta levels and rescues early cognitive deficit by increasing proteasome activity. Brain Pathol.

[43] Yuan L, Han J, Meng Q, Xi Q, Zhuang Q, Jiang Y (2015). Muscle-specific E3 ubiquitin ligases are involved in muscle atrophy of cancer cachexia: an in vitro and in vivo study. Oncol Rep.

[44] Zhao J, Brault JJ, Schild A, Cao P, Sandri M, Schiaffino S (2007). FoxO3 coordinately activates protein degradation by the autophagic/lysosomal and proteasomal pathways in atrophying muscle cells. Cell Metab.

[45] Masiero E, Agatea L, Mammucari C, Blaauw B, Loro E, Komatsu M (2009). Autophagy is required to maintain muscle mass. Cell Metab.

[46] Ju JS, Varadhachary AS, Miller SE, Weihl CC (2010). Quantitation of “autophagic flux” in mature skeletal muscle. Autophagy.

[47] Mizushima N, Yamamoto A, Matsui M, Yoshimori T, Ohsumi Y (2004). In vivo analysis of autophagy in response to nutrient starvation using transgenic mice expressing a fluorescent autophagosome marker. Mol Biol Cell.

[48] Salgo MG, Pryor WA (1996). Trolox inhibits peroxynitrite-mediated oxidative stress and apoptosis in rat thymocytes. Arch Biochem Biophys.

[49] Wu TW, Hashimoto N, Wu J, Carey D, Li RK, Mickle DA (1990). The cytoprotective effect of Trolox demonstrated with three types of human cells. Biochem Cell Biol.

[50] Drey M, Krieger B, Sieber CC, Bauer JM, Hettwer S, Bertsch T (2014). Motoneuron loss is associated with sarcopenia. J Am Med Dir Assoc.

[51] Edstrom E, Ulfhake B (2005). Sarcopenia is not due to lack of regenerative drive in senescent skeletal muscle. Aging Cell.

[52] Larsson L (1995). Motor units: remodeling in aged animals. J Gerontol A Biol Sci Med Sci.

[53] Walsh ME, Bhattacharya A, Liu Y, Van RH (2015). Butyrate prevents muscle atrophy after sciatic nerve crush. Muscle Nerve.

[54] Mammucari C, Milan G, Romanello V, Masiero E, Rudolf R, Del PP (2007). FoxO3 controls autophagy in skeletal muscle in vivo. Cell Metab.

[55] Mancinelli R, Kern H, Fulle S, Carraro U, Zampieri S, La Rovere R (2011). Trascriptional profile of denervated vastus lateralis muscle derived from patients 8 months after spinal cord injury: a case report. Int J Immunopathol Pharmacol.

[56] Gomes AV, Waddell DS, Siu R, Stein M, Dewey S, Furlow JD (2012). Upregulation of proteasome activity in muscle RING finger 1-null mice following denervation. FASEB J.

[57] Pigna E, Berardi E, Aulino P, Rizzuto E, Zampieri S, Carraro U (2016). Aerobic exercise and pharmacological treatments counteract cachexia by modulating autophagy in colon cancer. Sci Rep.

[58] Caron AZ, Haroun S, Leblanc E, Trensz F, Guindi C, Amrani A (2011). The proteasome inhibitor MG132 reduces immobilization-induced skeletal muscle atrophy in mice. BMC Musculoskelet Disord.

[59] Jamart C, Raymackers JM, Li AG, Deldicque L, Francaux M (2011). Prevention of muscle disuse atrophy by MG132 proteasome inhibitor. Muscle Nerve.

[60] Supinski GS, Vanags J, Callahan LA (2009). Effect of proteasome inhibitors on endotoxin-induced diaphragm dysfunction. Am J Physiol Lung Cell Mol Physiol.

[61] Enrico O, Gabriele B, Nadia C, Sara G, Daniele V, Giulia C (2007). Unexpected cardiotoxicity in haematological bortezomib treated patients. Br J Haematol.

[62] Grumati P, Coletto L, Sabatelli P, Cescon M, Angelin A, Bertaggia E (2010). Autophagy is defective in collagen VI muscular dystrophies, and its reactivation rescues myofiber degeneration. Nat Med.

[63] Vainshtein A, Desjardins EM, Armani A, Sandri M, Hood DA (2015). PGC-1alpha modulates denervation-induced mitophagy in skeletal muscle. Skelet Muscle.

[64] Friel JK, Friesen RW, Harding SV, Roberts LJ (2004). Evidence of oxidative stress in full-term healthy infants. Pediatr Res.

[65] Fulle S, Di DS, Puglielli C, Pietrangelo T, Beccafico S, Bellomo R (2005). Age-dependent imbalance of the antioxidative system in human satellite cells. Exp Gerontol.

[66] Labunskyy VM, Gladyshev VN (2013). Role of reactive oxygen species-mediated signaling in aging. Antioxid Redox Signal.

[67] Dobrowolny G, Aucello M, Rizzuto E, Beccafico S, Mammucari C, Boncompagni S (2008). Skeletal muscle is a primary target of SOD1G93A-mediated toxicity. Cell Metab.

[68] Muller FL, Song W, Jang YC, Liu Y, Sabia M, Richardson A (2007). Denervation-induced skeletal muscle atrophy is associated with increased mitochondrial ROS production. Am J Physiol Regul Integr Comp Physiol.

[69] Powers SK, Kavazis AN, DeRuisseau KC (2005). Mechanisms of disuse muscle atrophy: role of oxidative stress. Am J Physiol Regul Integr Comp Physiol..

[70] Sinha-Hikim I, Sinha-Hikim AP, Parveen M, Shen R, Goswami R, Tran P (2013). Long-term supplementation with a cystine-based antioxidant delays loss of muscle mass in aging. J Gerontol A Biol Sci Med Sci.

[71] Song L, Chen L, Zhang X, Li J, Le W (2014). Resveratrol ameliorates motor neuron degeneration and improves survival in SOD1(G93A) mouse model of amyotrophic lateral sclerosis. Biomed Res Int.

[72] Vays VB, Eldarov CM, Vangely IM, Kolosova NG, Bakeeva LE, Skulachev VP (2014). Antioxidant SkQ1 delays sarcopenia-associated damage of mitochondrial ultrastructure. Aging (Albany NY).

[73] Forni PE, Scuoppo C, Imayoshi I, Taulli R, Dastrù W (2006). High levels of Cre expression in neuronal progenitors cause defects in brain development leading to microencephaly and hydrocephaly. J Neurosci.

[74] Higashi AY, Ikawa T, Muramatsu M, Economides AN, Niwa A (2009). Direct hematological toxicity and illegitimate chromosomal recombination caused by the systemic activation of CreERT2. J Immunol.

[75] Shi J, Petrie HT (2012). Activation kinetics and off-target effects of thymus-initiated cre transgenes. PLoS One.

[76] Lexow J, Poggioli T, Sarathchandra P, Santini MP, Rosenthal N (2013). Cardiac fibrosis in mice expressing an inducible myocardial-specific Cre driver. Dis Model Mech.

[77] Pugach EK, Richmond PA, Azofeifa JG, Dowell RD, Leinwand LA (2015). Prolonged Cre expression driven by the α-myosin heavy chain promoter can be cardiotoxic. J Mol Cell Cardiol.

