# Supplementary material for: HDAC4 preserves skeletal muscle structure following long-term denervation by mediating distinct cellular responses
Source: Skelet Muscle. 2018 Feb 24;8:6. doi: 10.1186/s13395-018-0153-2 (PMC6389241; doi:10.1186/s13395-018-0153-2)
Supplement: Supplementary file 1 — Figure S1. Denervation differentially affected HDAC4mKO and control mice. Figure S2. HDAC4mKO muscles did not show differences in dystophin glycoprotein complex. Figure S3. Methylene blue and intermittent fasting efficiently activated UPS and autophagy in HDAC4mKO mice, respectively. Figure S4. Effects of methylene blue and intermittent fasting on HDAC4mKO muscles. Figure S5. HDAC4mKO mice showed altered levels of Gp91phox upon denervation. Figure S6. Trolox treatment efficiently reduces free radical levels in HDAC4mKO mice. (DOC 3765 kb) [file 13395_2018_153_MOESM1_ESM.doc]

**Additional file**

**Figure S1** Denervation differentially affected HDAC4mKO and control mice. **a** Weight of HDAC4mKO and control (CTR) denervated TA muscles, over contralateral ones, expressed as the percentage, over time following denervation. Data are shown as mean ± SEM; n=4; two-way ANOVA (F=4.53; df 1; p=0.043) revealed an interaction between the genotypes and treatment (denervation); *p<0.05 by Tukey’s HSD test. **b** Laminin staining of control (CTR) and HDAC4mKO TA muscles following one, two and four weeks of denervation. Scale bar=50 micron.

**Figure S2** HDAC4mKO muscles did not show differences in dystophin glycoprotein complex. Representative images of immunofluorescence for dystophin or alpha-dystroglycan in HDAC4mKO and control TA muscles, following four weeks of denervation. Scale bar=50 micron.

**Figure S3** Methylene blue and intermittent fasting efficiently activated UPS and autophagy in HDAC4mKO mice, respectively. **a** Proteasome activity in HDAC4mKO TA muscles, without (-) or with MB treatment, two weeks following denervation. Data are shown as mean ± SEM; n=3-4. Two-way ANOVA (F=10; df 1; p=0.01) revealed an interaction between treatments (denervation and MB); *p<0.05; by Tukey’s HSD test. **b** Western blot analyses for Myosin Heavy Chain (MHC) in contralateral and denervated HDAC4mKO muscles, two weeks following denervation, without (-) or with MB treatment. **c** Representative images of HDAC4mKO TA muscles co-electroporated with Ub-G76V-YFP (green) and dsRED (red) plasmids, without (-) or with MB treatment, and relative quantification. Scale bar=50 micron. n=3; between 45 and 300 dsRED^+^ fibers were counted per each sample. Two-way ANOVA (F=8.56; df 1; p=0.02) revealed an interaction between treatments (denervation and MB); *p<0.05; by Tukey’s HSD test. **d** Real-time PCR for autophagic markers in HDAC4mKO skeletal muscle, one week following denervation, in the absence (-) or IF. Data are shown as mean ± SEM; n=8. Two-way ANOVA revealed an effect of the treatment (IF) (F=29.3; df 1; p=0.0001 for Atg7, F=19.34; df 1; p=0.0003 for Gabarapl1, F=9.65; df 1; p=0.0045 for Atg5) and an interaction between untreated and treated denervated HDAC4mKO muscles; *p<0.05 by Tukey’s HSD test. **e** Densitometric measurements of western blot analyses for LC3b and p62 proteins, one week following denervation, in the absence (-) or after IF treatment. Gapdh was used as loading control. Data are shown as mean ± SEM; n=3-4. Two-way ANOVA revealed an effect of the treatment (IF) (F=5.50; df 1; p=0.036 for LC3b, F=22.93; df 1; p=0.0006 for p62) and an interaction between untreated and treated denervated HDAC4mKO muscles; *p<0.05 by Tukey’s HSD test.

**Figure S4** Effects of methylene blue and intermittent fasting on HDAC4mKO muscles. **a** Distribution analysis of HDAC4mKO fiber CSA, following 4 weeks of denervation, without or with MB treatment. Data are shown as mean ± SEM; n=4; two-way ANOVA (F=7.27; df 1; p=0.026 and F=7.27; df 1; p=0.026) revealed an interaction between treatments (denervation and MB) in 1000-1500 µm^2^ and 2500-3000 µm^2^ cross-sectional area classes, respectively; **p<0.01 by Tukey’s HSD test. **b** Distribution analysis of HDAC4mKO fiber cross-sectional area, following 4 weeks of denervation, without or with IF treatment. Data are shown as mean ± SEM; n=4; two-way ANOVA (F=10.2; df 1; p=0.01 for 1000-1500 µm^2^ class and F=8.2; df 1; p=0.01 for 2500-3000 µm^2^ class) revealed an interaction between treatments (denervation and IF); **p<0.01 by Tukey’s HSD test. **c** Weight of HDAC4mKO muscles without (-) or after MB or IF treatment, over contralateral (-) ones. n=4-5; two-way ANOVA (F=103.5; df 1; p=0.001 for MB and F=40; df 1; p=0.0001 for IF) revealed an effect of denervation without interaction between treatments; *p<0.05 by Tukey’s HSD test.

**Figure S5** HDAC4mKO mice showed altered levels of Gp91phox upon denervation. Representative western blot analyses for Gp91phox in control (CTR) and HDAC4mKO muscle after **a** one, **b** two, and **c** four weeks following denervation. **d** Real-time PCR of antioxidant enzymes in control (CTR) and HDAC4mKO skeletal muscles, four week following denervation. Data are shown as mean ± SEM; n=4. Two-way ANOVA (F=7.97; df 1; p=0.018 for catalase) revealed an effect of denervation; *p<0.05 by Tukey’s HSD test.

**Figure S6** Trolox treatment efficiently reduces free radical levels in HDAC4mKO mice. **a** DHE staining of HDAC4mKO skeletal muscle following four weeks of denervation without (-) or after Trolox (TRX) treatment and relative quantification. Scale bar=50 micron. n=3; two-way ANOVA (F=209; df 1; p=0.0001) showed an interaction between treatments (denervation and Trolox); *p<0.05 by Tukey’s HSD test. **b** Quantification of free radical levels (ROS and RNS) in HDAC4mKO skeletal muscle following four weeks of denervation without (-) or after Trolox treatment. n=3; two-way ANOVA (F=14.4; df 1; p=0.0053) revealed an effect of denervation.

S1


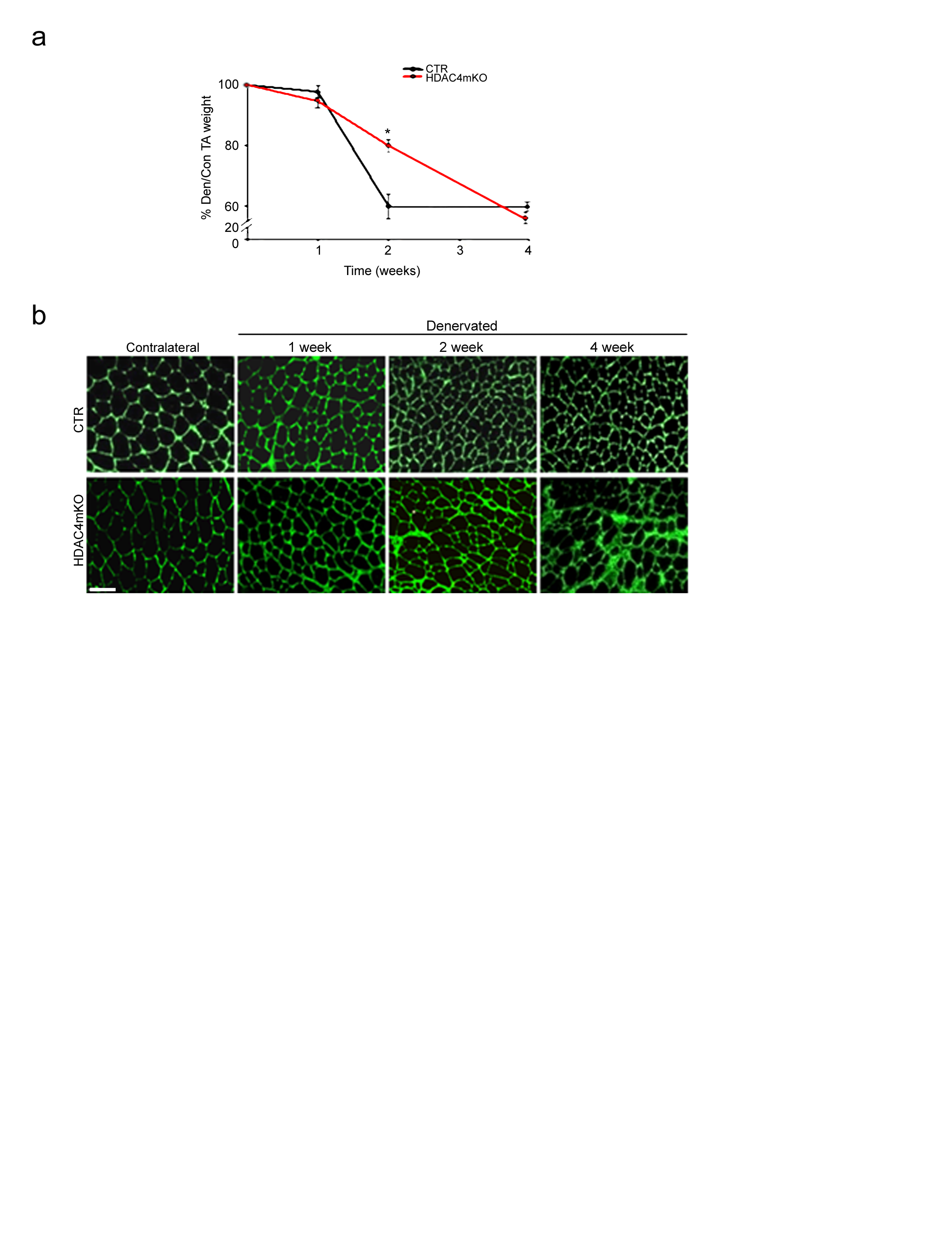


S2


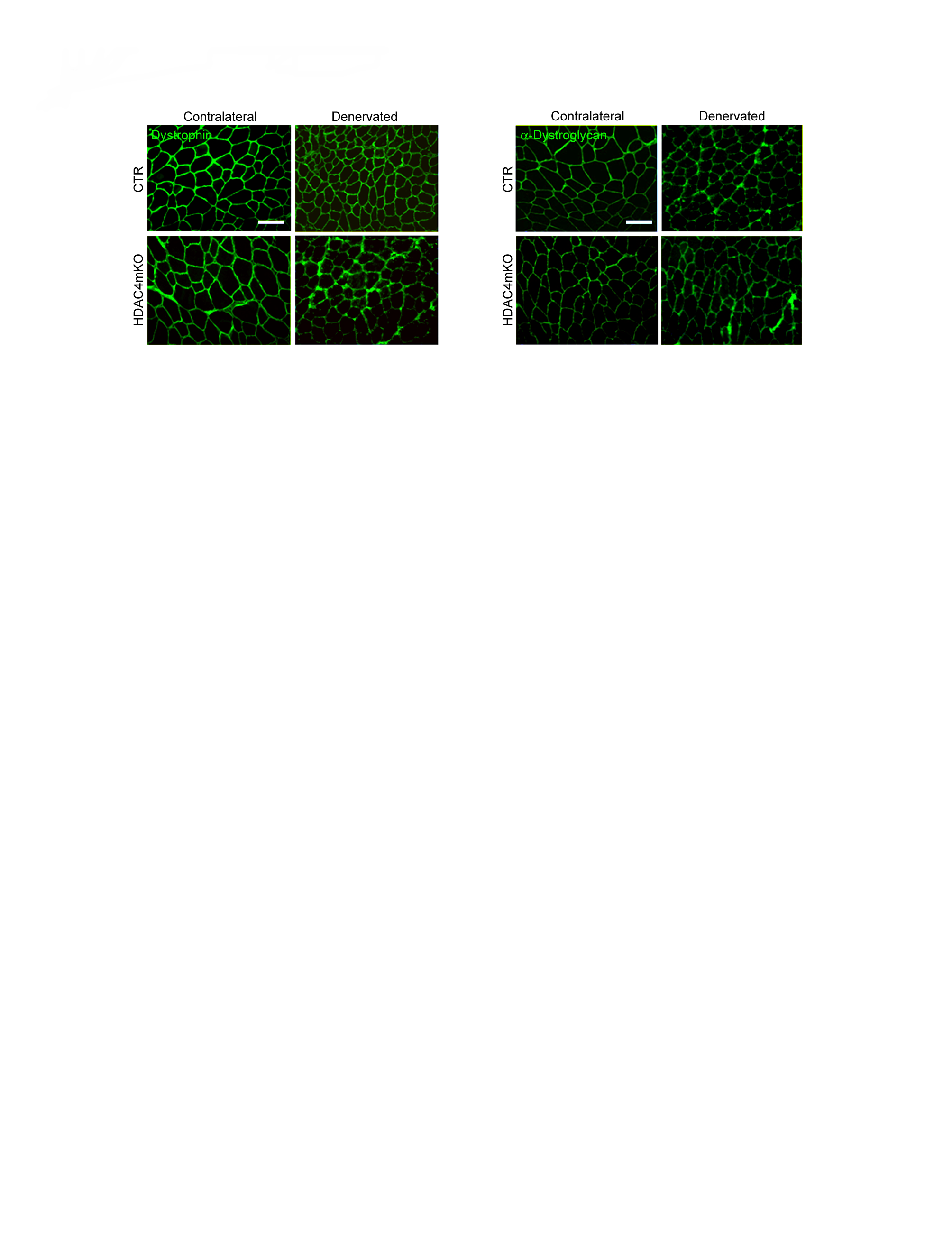


S3


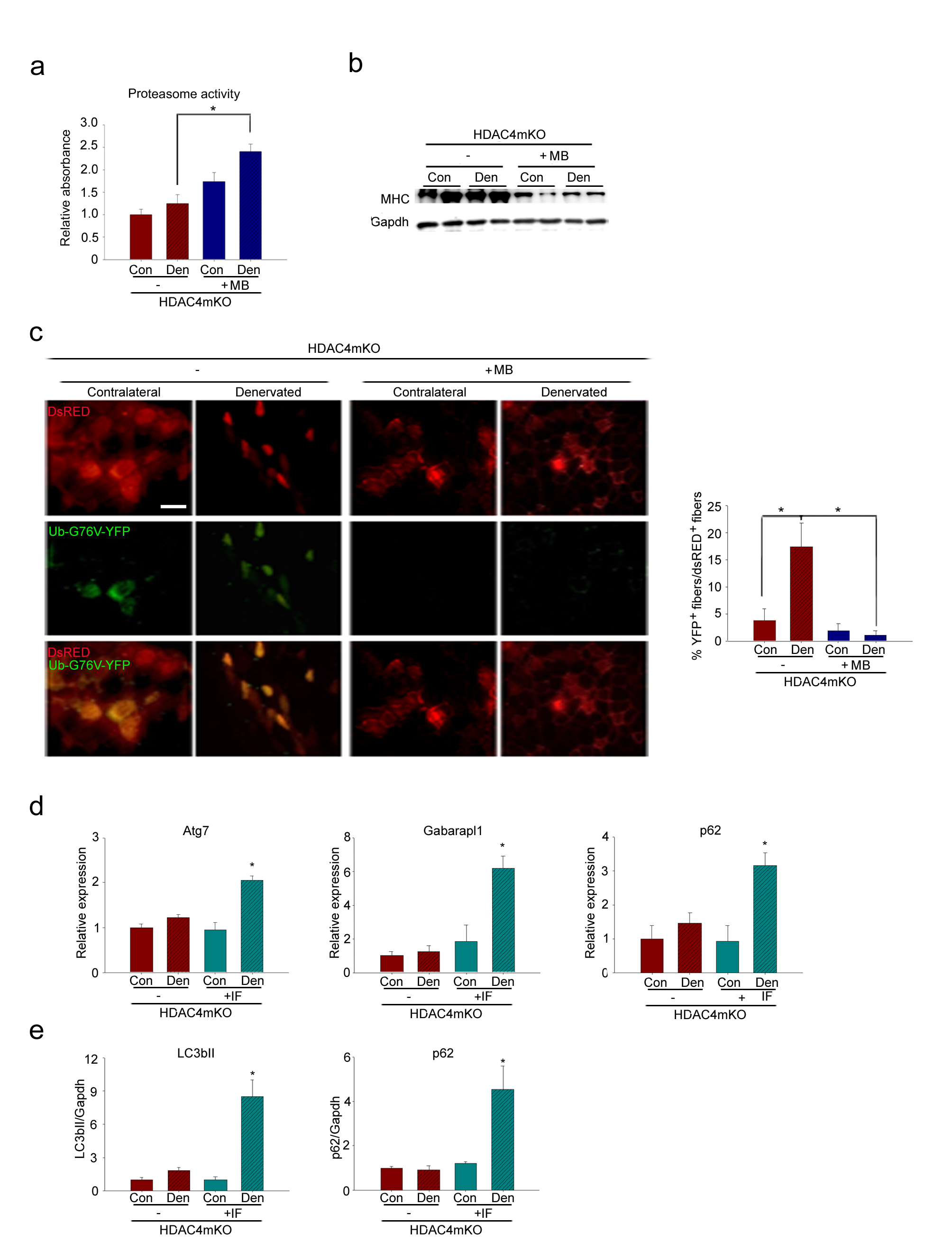


S4


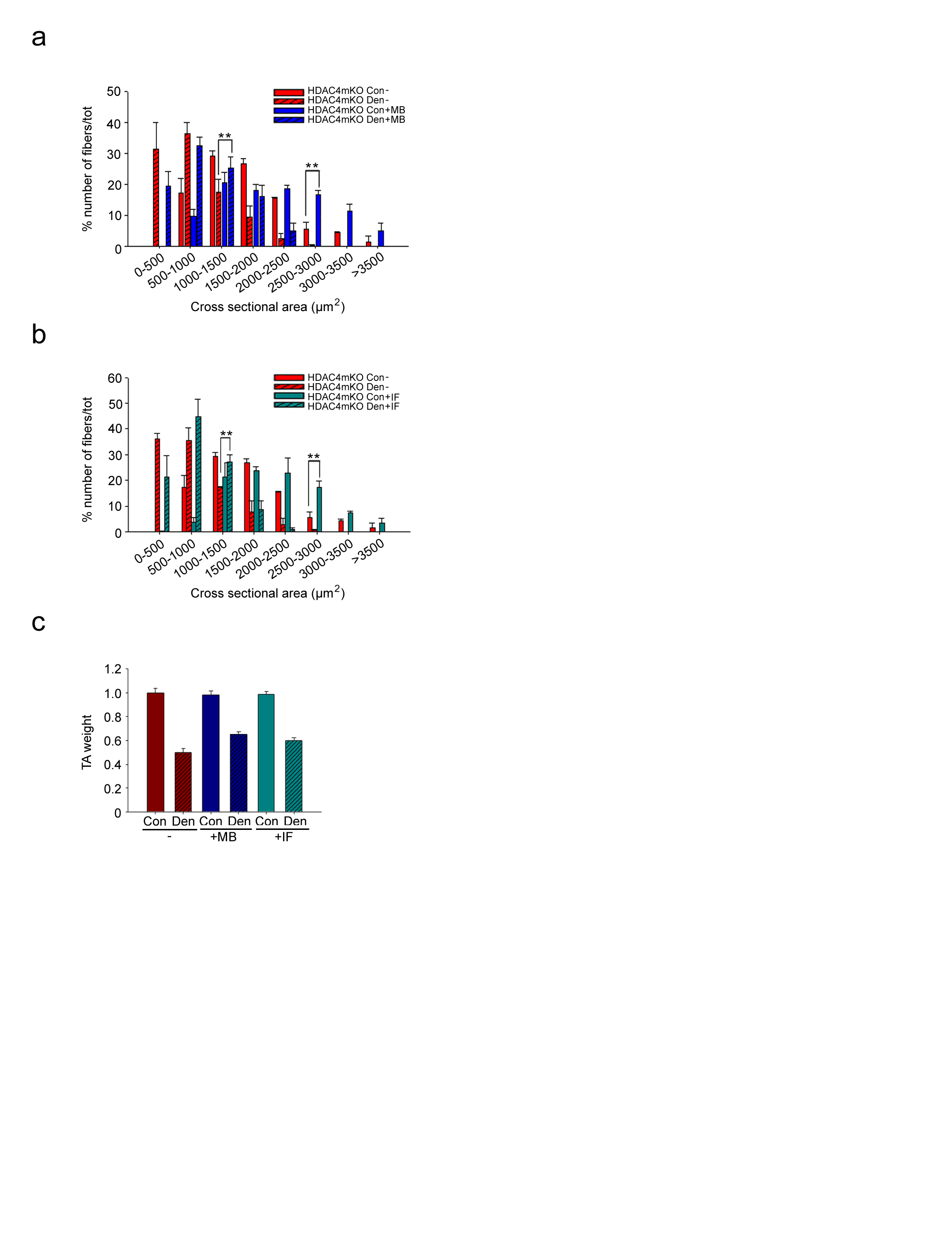


S5
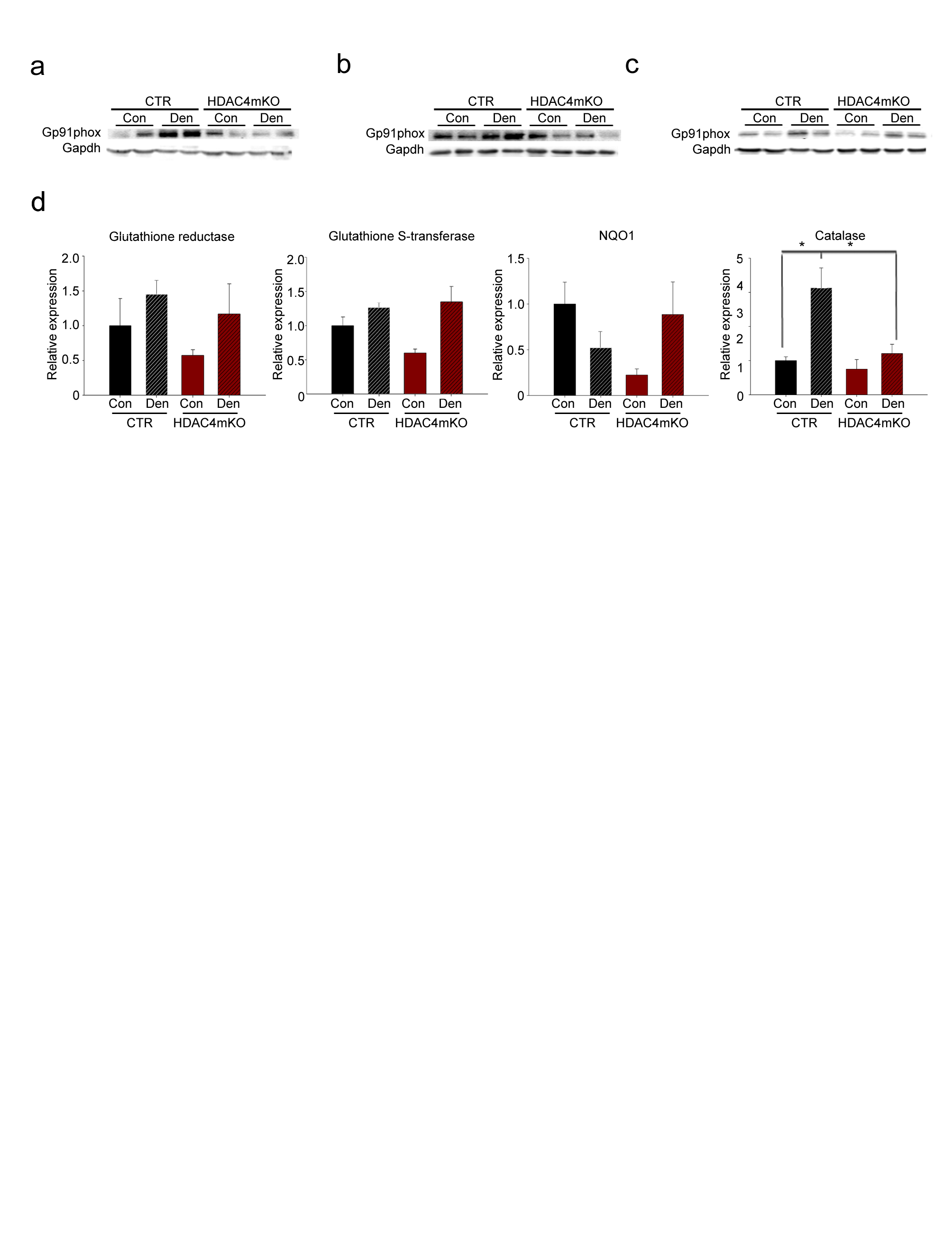


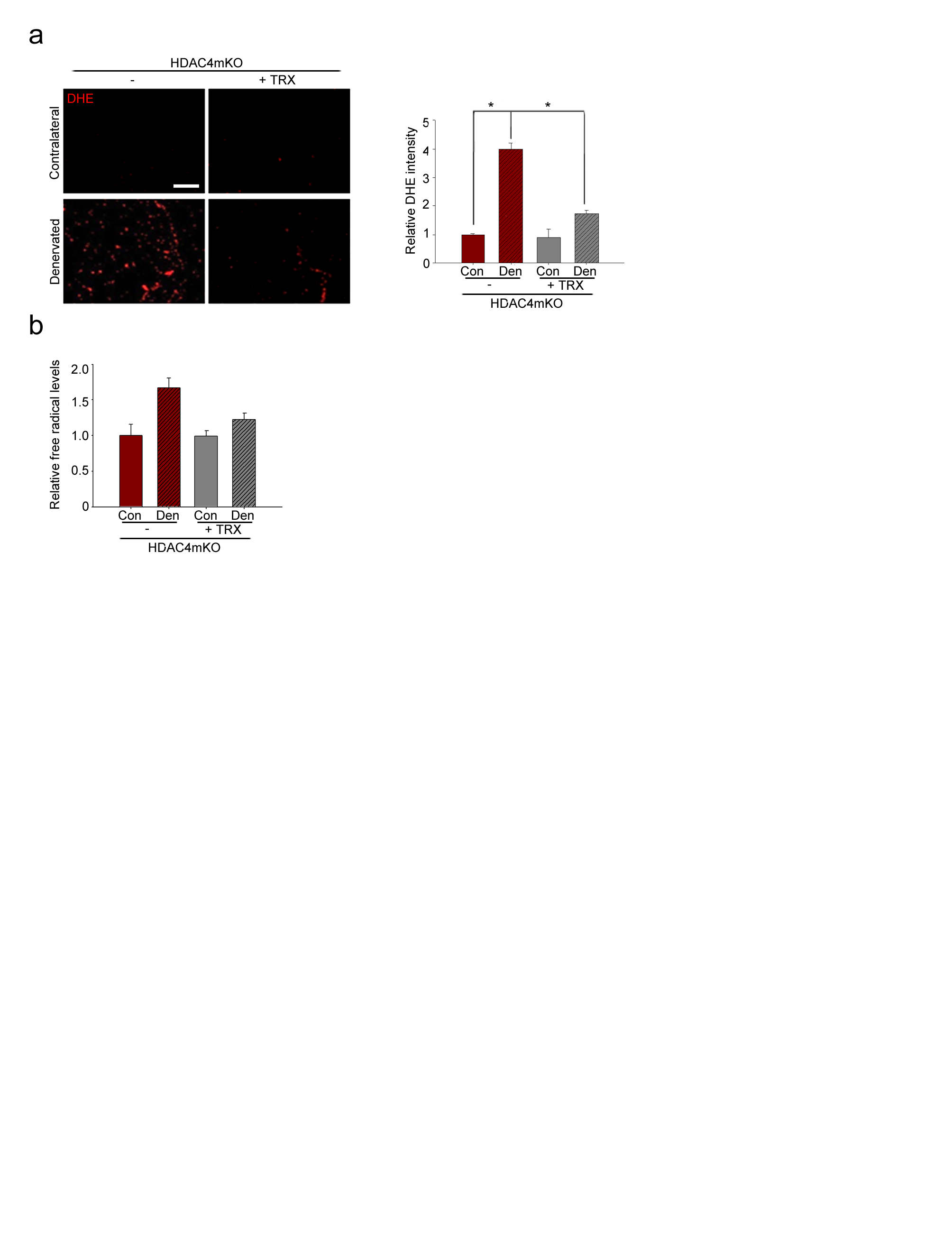
S6
